# Evaluating the N1-P2 Interpeak Latency of the eCAP and Its Intertrial Variability as Potential Indicators of Neural Synchrony in the Auditory Nerve of Cochlear Implant Users

**DOI:** 10.1007/s10162-026-01051-1

**Published:** 2026-05-11

**Authors:** Shuman He, Ian C. Bruce, Zi Gao, Ross A. Aiello, Christopher R. Mueller

**Affiliations:** 1 Department of Otolaryngology–Head and Neck Surgery, The Ohio State University, 915 Olentangy River Road, Columbus, OH 43212 USA; 2 Department of Electrical and Computer Engineering, McMaster University, Hamilton, ON L8S 4K1 Canada; 3 Department Mechanic Engineering & Material Science, Washington University in St. Louis, St. Louis, MO 63105 USA

**Keywords:** Electrical stimulation, Auditory nerve, Neural synchrony, Interpeak latency

## Abstract

**Objective:**

This study evaluated interpeak latency (IPL) and its intertrial variability (IPLVAR) of the electrically evoked compound action potential (eCAP) as potential alternatives to the phase-locking value (PLV) for quantifying auditory nerve (AN) synchrony in cochlear implant (CI) users.

**Design:**

The IPL and IPLVAR were assessed in 28 postlingually deafened adults, 37 children with auditory neuropathy spectrum disorder (ANSD), 43 children with cochlear nerve deficiency, and 38 children with typical sensorineural hearing loss. All participant groups include both male and female participants. Their associations with temporal resolution and speech perception outcomes were evaluated in children with ANSD and/or adult CI users. Frequency analysis was conducted to understand the impacts of eCAP recording noise on the IPL, IPLVAR, and PLV. Simulations of intertrial jitter in the eCAP were performed to quantify how the IPL, IPLVAR, and PLV metrics varied with increased temporal jitter and its interaction with recording noise level and sampling rate.

**Results:**

eCAP traces recorded in all patient groups showed a multipoint peak issue affecting the accuracy of IPL and IPLVAR assessments. Temporal resolution and speech perception outcomes were significantly correlated with IPLVAR but not with IPL metrics. The PLV was impacted less by recording noise than either the IPL or the IPLVAR. Simulation results revealed that the IPL was less sensitive to the amount of intertrial jitter in the eCAP than were the IPLVAR and the PLV.

**Conclusions:**

The IPL is not a reliable indicator of AN synchrony. The IPLVAR is indicative of neural synchrony in the AN; however, it is more strongly affected by high levels of eCAP recording noise than the PLV. The PLV is therefore the preferred measure for quantifying neural synchrony in the AN in CI users.

**Supplementary Information:**

The online version contains supplementary material available at 10.1007/s10162-026-01051-1.

## Introduction

The ability of human listeners to detect, discriminate, and recognize complex auditory signals depends on temporally organized neural activity in the auditory system that is time-locked to the auditory input (i.e., neural synchrony) (e.g., [1–9]). For cochlear implant (CI) users, the auditory nerve (AN) is the first neural structure to receive electrical stimulation from the device. Consequently, the fidelity with which the AN encodes and processes this stimulation critically influences both the quality and quantity of auditory information conveyed to higher-order auditory centers. In animal models, the discharge patterns of AN fibers have been shown to play a key role in encoding temporal cues [10–12]. Consistent with these findings from single-fiber recordings, simulation studies using computational models have demonstrated that poor neural synchrony in the AN results in a degraded neural representation of temporal envelope cues, leading to deficits in the perception of these cues [1, 2]. Given the limited availability of temporal fine structure cues and the reduced spectral resolution provided by current CIs [13, 14], CI users primarily depend on temporal envelope cues for speech understanding (e.g., [15, 16]). Consequently, neural synchrony in the AN is expected to play a critical role in determining speech perception outcomes in this population. However, the lack of noninvasive methods for assessing AN neural synchrony has historically hindered its investigation in CI users.

Recently, He et al. [17] reported the use of the phase-locking value (PLV) to assess neural synchrony in the AN in CI patients. In this method, the PLV is calculated based on trial-to-trial phase coherence in the summated activity of AN fibers evoked by electrical stimulation (i.e., the electrically evoked compound action potential, eCAP). Their findings demonstrated the feasibility of applying this method in both adult and pediatric CI populations [17–19], including children with auditory neuropathy spectrum disorder (ANSD). Moreover, consistent with the literature on acoustic hearing [1, 2], PLV was found to correlate with temporal resolution and speech perception in noise in postlingually deafened adult CI users [17, 18]. These results suggest that PLV holds strong promise as an objective indicator of perceptual outcomes in CI users. However, as noted by He et al. [17], this method is affected by the sampling rate (F_s_) of CI devices, which varies substantially across CI manufacturers. The extent to which this variability affects PLV measurements remains unclear, posing a challenge for studies involving participants with different CI systems.

As part of the effort to develop a noninvasive method for assessing neural synchrony in the AN in human CI users, we evaluated, in addition to the PLV, the intertrial variability of the eCAP interpeak latency (IPLVAR) as a potential alternative indicator of neural synchrony [20]. More recently, Schvartz-Leyzac et al. [21] proposed that the interpeak latency (IPL) of the average eCAP waveform could potentially serve as a marker of neural synchrony. The selection of these two metrics was guided by findings in the acoustic hearing literature, which demonstrate that decreased neural synchrony is associated with delayed response peaks and increased intertrial variability [22–26]. Both indicators are based on the morphological characteristics of the eCAP. As a result, their computation does not depend on manufacturer-specific parameters. Therefore, if the validity of the IPL and the IPLVAR as indicators of AN neural synchrony is established, they can effectively address a key limitation of the PLV method.

In the present study, the IPL was defined as the difference between the N1 and P2 latencies of the eCAP waveform for a given electrode in each participant. The IPLVAR was defined as the standard deviation of the corresponding IPL of the individual eCAP sweeps (i.e., trials) measured at the same electrode in the same participant. For healthy AN fibers, spikes elicited by direct electrical stimulation at the single-unit level exhibit considerably less temporal jitter compared to those evoked by synaptic inputs from hair cells [27–29]. However, axonal dystrophy and demyelination of AN fibers, a common issue observed in CI users (e.g., [30–36]), alter many neural properties (e.g., [37, 38]). These alterations lead to complex changes in spike timing [39] and action potential initiation sites (e.g., [39–42]). As a result, spike synchronizations within individual AN fibers across repeated stimulations as well as among different AN fibers are reduced, potentially resulting in delayed response peaks and increased intertrial variability of neural responses during electrical hearing. This scientific basis supports the use of the IPLVAR to quantify temporal jitter of individual AN fibers, as measured using single-fiber recordings in animal models, where smaller IPLVAR values indicate less temporal jitter and thus better neural synchrony [27]. However, as shown by simulation results reported in Resnick et al. [39], the effects of demyelination on fiber sensitivity and spike timing at the single-unit level are complex and highly heterogeneous. Furthermore, these effects strongly depend on the degree of demyelination, which has been shown to vary substantially along the cochlea, even within individual CI patients [36]. As a near-field recorded compound response in CI patients, the eCAP is not only affected by neural factors (e.g., neural synchrony, discharge rate of individual AN fibers, neural survival of the AN) but also affected by nonneural factors (e.g., impedance, electrode-to-modiolus distance). This distinction is important compared to the far-field recorded compound action potential in listeners with acoustic hearing. Therefore, rather than directly extrapolating findings from acoustic hearing to CI patients, it is essential to empirically evaluate whether reduced spike synchronization observed at the single-unit level translates into measurable changes in the IPL and IPLVAR in human CI users. This evaluation is a critical, scientifically rigorous prerequisite before applying these metrics in clinical and research contexts involving CI populations.

Evaluations were conducted by (1) comparing results across four patient populations with different pathophysiological conditions affecting the AN and (2) assessing the associations between the IPL, the IPLVAR, temporal resolution, and speech perception outcomes in pediatric CI users with ANSD and/or postlingually deafened adult CI users. The rationale for this approach is that a robust indicator needs to be sensitive to the pathophysiological condition it probes, as well as predictive of the resulting auditory perception outcomes. The first patient population consisted of children with ANSD, a condition characterized by poor neural synchrony that cannot be fully compensated for by the enhanced neural synchronization provided by electrical stimulation [1, 43]. The second patient population included children with cochlear nerve deficiency (CND), a group with substantially fewer but otherwise healthy AN fibers compared to children with idiopathic sensorineural hearing loss (SNHL) and normal-sized ANs [44, 45]. The third patient population was children with typical SNHL. The fourth patient population was postlingually deafened adult CI users, who exhibit greater loss of spiral ganglion somas and peripheral processes at the cochlear base relative to the apex [35, 46].

Data from all four patient populations were analyzed to evaluate the IPL and the IPLVAR. Additionally, relationships between the PLV and these two indicators were examined across all four patient groups, and associations between age and these two indicators were assessed in the adult cohort. A frequency analysis of eCAP recording noise was performed to determine how the noise spectrum impacts the frequency-domain PLV metric compared to the time-domain IPL and IPLVAR measures. Finally, computational modeling was employed to understand the effect of the amount of temporal jitter, recording noise, and sampling rate on IPL, IPLVAR, and PLV metrics. Based on the literature reviewed previously, shorter IPLs, smaller IPLVARs, and larger PLVs were expected to be indicative of stronger neural synchrony in the AN.

We hypothesized that reduced spike synchronization within and across AN fibers would lead to greater intertrial variability of the eCAP, which can be quantified using the IPLVAR. This study also tested the assumption made by Schvartz-Leyzac et al. [21] regarding the effect of reduced neural synchrony on the IPL. Children with ANSD were expected to show longer IPLs and larger IPLVARs than children with typical SNHL and children with CND. Among adult CI users, longer IPLs and larger IPLVARs were predicted at basal electrode locations compared to more apical sites. Furthermore, longer IPLs and larger IPLVARs were anticipated to correlate with poorer temporal resolution and lower speech perception scores in noise. Finally, strong correlations were expected among IPL, IPLVAR, and PLV metrics measured within the same patient cohort.

## Materials and Methods

### Overview

This study comprised three experiments conducted in patients implanted with Cochlear™ Nucleus® devices (Cochlear Ltd., Macquarie, NSW, Australia). The first and second experiments evaluated the IPLVAR and IPL, respectively, while the third experiment assessed the recording noise associated with eCAP measurements in current Cochlear™ Nucleus® devices. The electrode array of Cochlear™ Nucleus® devices contains 22 electrodes, with those located toward the tip of the array assigned larger numbers. Consequently, electrode 22 is positioned at a more apical, intracochlear location than electrode 1 in Cochlear™ Nucleus® device users.

These three experiments were approved by the biomedical Institutional Review Board (IRB) of The Ohio State University (IRB study # 2017H0131, 2018H0344, and 2018N0005) and the IRB of the University of North Carolina at Chapel Hill (IRB study # 12–1737). Informed consent was obtained from all participants included in this study.

### Datasets

#### Datasets of Experiment 1 Evaluating the IPLVAR

Calculation of the IPLVAR was based on results from individual eCAP sweeps. These analyses utilized three previously published datasets: one from a study involving children with ANSD [19] and two from studies involving postlingually deafened adult CI users [17, 18], along with newly collected data from four ears of three children with ANSD (one girl and two boys), 15 ears of 13 children with CND (six girls and seven boys), and 15 ears of 13 children with typical SNHL (seven girls and six boys).

These datasets were used (1) to compare IPLVARs across electrode locations in the three pediatric participant groups, (2) to compare IPLVARs across four electrode locations within the adult participant group, (3) to assess the associations between the IPLVAR and behavioral gap detection threshold (GDT) and between the IPLVAR and speech perception scores in adult participants, and (4) to examine the associations between the PLV and the IPLVAR and between the PLV and the IPL across the four participant groups.

#### Datasets of Experiment 2 Evaluating the IPL

In Schvartz-Leyzac et al. [21], the IPL was calculated based on the average eCAP obtained from 50–100 sweeps. It should be noted that averaging eCAP responses across 50 or 100 sweeps is the default setting in the Custom Sound EP software (Cochlear Ltd., Macquarie, NSW, Australia) which is commonly used in the literature. With this default setting, only the averaged eCAP response is saved, whereas individual eCAP sweeps are not retained in the data files. To ensure comparability with previous studies, IPL evaluations in the present work utilized three previously published datasets collected across three studies involving children with ANSD, CND, and SNHL, all of which were collected using this default setting [43, 47, 48]. There was no overlap between the pediatric participants included in these datasets and those in Experiment 1. The same two datasets from postlingually deafened adult CI users [17, 18] used in Experiment 1 were also included in the IPL analysis.

These datasets were used (1) to compare IPLs across electrode locations in the three pediatric participant groups, (2) to compare IPLs across four electrode locations within the adult participant group, and (3) to assess the associations between the IPL and GDT and between the IPL and speech perception scores in children with ANSD and adult participants.

#### Dataset for Evaluating the Effect of Age in the Adult Cohort

Schvartz-Leyzac et al. [21] reported a significant age effect on the IPL. To facilitate comparison with their findings, the effects of age on the IPLVAR and the IPL were evaluated using the same adult dataset used in both Experiments 1 and 2.

#### Datasets of Experiment 3 Evaluating the Recording Noise

The evaluation was conducted using another new dataset obtained from a subgroup of 16 adult CI users (17 ears: A003L, A006L, A008L, A008R, A009L, A020R, A024R, A035L, A052L, A061R, A064L, A070R, A075R, A077L, A080L, A091L, and A093R) who also participated in the studies reported in Gao et al. [18] and/or He et al. [17]. These data were utilized in a frequency analysis to assess the likely impact of eCAP recording noise on IPL, IPLVAR, and PLV metrics. In addition, these data were used to estimate the IPL, the IPLVAR, and the PLV at the noise floor. Although these analyses were not central to the primary research questions of this study, they provide critical reference values for interpreting physiological IPL, IPLVAR, and PLV measurements in Cochlear™ Nucleus® device users, which are currently absent from the literature.

For the existing datasets, participant demographic information and the electrode locations tested in the different experiments have been reported in the previously cited studies. Therefore, Table 1 provides a summary of the datasets included in each experiment, basic demographic information for each participant group, and the parameters used for eCAP recordings. Detailed demographic information for the 29 children newly tested in Experiment 1 is presented in Table 2.

**Table 1:** Summary of datasets included in different experiments, basic demographic information for each participant group, and parameters used for electrically evoked compound action potential (eCAP) recordings

Experiment	Dataset				Participant age (year)	eCAP parameters				
	Patient group	Results included	Sample size (ear)	Previous study based on the dataset		Electrode number of default electrode location	Pulse phase duration (μs)	Recording window (μs)	System gain	Number of sweeps
Experiment 1	ANSD	eCAP IPLVAR and PLV	15	He et al. (2024)	3.30–14.30 (mean, 7.28; SD, 3.28)	3, 12, 19	25–62	1600	50	400
	SNHL	eCAP IPLVAR and PLV	15	N/A	1.98–14.66 (mean, 9.70; SD, 4.13)	3, 13, 19	25–37	1600	50	400
	CND	eCAP IPLVAR and PLV	15	N/A	1.80–15.85 (mean, 6.75; SD, 3.85)	No default	50	1600	40	400
	Adult	Age, eCAP IPLVAR, PLV, and AzBio scores	28	Gao et al. (2025)	20.39–84.04 (mean, 61.86; SD, 14.78)	3, 9, 15, 21	25–37	1600	50	400
		GDT	15	He et al. (2024)	36.58–84.01 (mean, 62.92; SD, 13.88)	No default	25–37			
Experiment 2	ANSD	eCAP IPL	30	He et al. (2016)	2.8–17.8 (mean, 8.1; SD, 3.2)	3, 12, 21	25–37	1600	50	50
		GDT and PBK scores	10	He et al. (2013)	5.30–17.20 (mean, 8.34; SD, 3.25)	12	25			
	SNHL	eCAP IPL	29	He et al. (2016)	2.8–17.8 (mean, 8.2; SD, 3.8)	3, 12, 21	25	1600	50	50
	CND	eCAP IPL	30	He et al. (2020)	2.14–8.95 (mean, 4.65; SD, 2.14)	No default	50	1600	40	100
	Adult	eCAP IPL and AzBio scores	28	Gao et al. (2025)	20.39–84.04 (mean, 61.86; SD, 14.78)	3, 9, 15, 21	25–37	1600	50	400
		GDT	15	He et al. (2024)	36.58–84.01 (mean, 62.92; SD, 13.88)	No default	25–37			
Experiment 3	Adult	Noise floor	17	N/A	36.79–84.04 (mean, 63.32; SD, 14.62)	3, 9, 15, 21		3200	50	400

*ANSD* auditory neuropathy spectrum disorder; *SNHL* sensorineural hearing loss; *CND* cochlear nerve deficiency; *IPLVAR* the intertrial variability of the eCAP interpeak latency; *IPL* interpeak latency; *PLV* phase-locking value; *PBK* Phonetically Balanced Kindergarten word test; *GDT* gap detection threshold; *N/A* not applicable; *SD* standard deviation

**Table 2:** Demographic information of the study participants newly tested in Experiment 1 of this study

Participant group	Participant ID	Ear tested	Age at testing (years)	Etiology	Internal device and electrode array	Electrode array type	Electrodes tested
Children with ANSD	ANSD1	L	5.2	OTOF	24RE (CA)	PM	3, 12, 21
	ANSD2	L	5.88	Unknown	CI632	PM	3, 12, 21
	ANSD2	R	5.87	Unknown	CI632	PM	3, 12, 21
	ANSD3	R	10.80	Unknown	CI632	PM	3, 12, 21
	CND01	L	3.86	Unknown	CI422	LW	3, 12, 21
Children with CND	CND02	R	4.47	Unknown	CI422	LW	3, 12, 21
	CND03	L	15.85	Unknown	24RE (CA)	PM	3, 12, 21
	CND04	L	5.46	Unknown	CI512	PM	3, 6, 12
	CND05	R	7.16	Unknown	CI522	LW	3, 12, 21
	CND06	L	2.09	Unknown	CI512	PM	3, 12, 21
	CND06	R	1.80	Unknown	CI512	PM	3, 12, 21
	CND07	L	6.94	Unknown	CI512	PM	3, 12, 21
	CND08	L	1.84	Unknown	CI512	PM	1, 4, 7
	CND09	L	9.67	Unknown	24RE (CA)	PM	3, 12, 21
	CND10	L	6.43	Unknown	24RE (CA)	PM	1, 6, 11
	CND11	R	8.52	Unknown	24RE (CA)	PM	3, 12, 21
	CND12	L	7.69	Unknown	24RE (CA)	PM	3, 12, 21
	CND12	R	7.69	Unknown	24RE (CA)	PM	3, 12, 21
	CND13	R	11.81	Unknown	24RE (CA)	PM	3, 12, 20
Children with typical SNHL	P33	L	14.66	Unknown	24RE (CA)	PM	3, 12, 21
	P33	R	14.66	Unknown	24RE (CA)	PM	3, 12, 21
	P56	L	9.94	Mondini	CI512	PM	3, 12, 20
	P62	L	13.17	Unknown	CI632	PM	3, 12, 21
	P63	L	5.11	SLC26A4	CI612	PM	3, 9, 21
	P64	L	12.85	Unknown	CI622	LW	6, 12, 21
	P75	R	4.44	MYO7A	CI512	PM	3, 12, 20
	P88	L	10.63	Unknown	CI632	PM	3, 12, 21
	P90	R	10.26	Unknown	CI532	PM	3, 12, 21
	P91	R	9.89	Unknown	CI632	PM	3, 12, 21
	P100	L	13.11	Unknown	24RE (CA)	PM	3, 12, 21
	P100	R	13.18	Unknown	24RE (CA)	PM	2, 12, 20
	P103	L	4.34	Unknown	CI632	PM	3, 12, 21
	P104	L	7.32	Unknown	CI522	LW	2, 12, 21
	P105	R	1.98	EVA	CI612	PM	3, 12, 21

*ANSD* auditory neuropathy spectrum disorder; *SNHL* sensorineural hearing loss; *CND* cochlear nerve deficiency; *L* left ear; *R* right ear; *CI24RE (CA)* Freedom Contour Advance electrode array; *PM* perimodiolar; *LW* lateral wall; *OTOF* otoferlin genetic mutation; *SLC24A4* solute carrier family 26 member 4 genetic mutation; *MYO7A* myosin VIIA genetic mutation; *EVA* enlarged vestibular aqueduct

### eCAP Recordings

For all participants, the stimulus used to elicit the eCAP was a cathodic-leading, charge-balanced biphasic pulse with an interphase gap of 7 μs. In Experiments 1 and 2, the stimulus was presented at the maximum comfortable level (i.e., C level) determined for each electrode, while in Experiment 3, it was presented at 0 clinical unit. eCAPs were recorded using the Custom Sound EP software (Cochlear Ltd., Macquarie, NSW, Australia) and a two-pulse, forward-masking paradigm with a masker-probe interval of 400 μs [49]. Due to the time required to acquire eCAP data using the Custom Sound EP software, collecting 400 eCAP sweeps required approximately 18 min, resulting in an effective stimulation rate of approximately 0.37 Hz. For all pediatric participants in Experiment 2, eCAPs were measured using a stimulation rate of 15 Hz.

In pediatric and adult CI users, eCAPs were recorded at three and four electrode locations across the electrode array, respectively. For children with CND, the three electrode locations extended to the most apical site at which an eCAP could be recorded, which varied across participants. As a result, there was no default electrode used for eCAP recording in this group. For children with CND, the selected electrodes were categorized as basal, middle, and apical based on their relative positions among the electrodes with measurable eCAPs. For all other participant groups, the default electrode numbers were used to indicate the electrode locations tested. In cases where an eCAP could not be obtained at the default electrode location, adjacent CI electrodes were tested. The default electrode locations, pulse phase duration, system gain, recording window duration, and number of sweeps included in the averaged eCAP response or retained in the saved data file varied across participant groups and experiments. These details are provided in Table 1. All eCAP results included in this study exhibited morphological characteristics consistent with prior reports [50] and showed no apparent evidence of electrical artifact contamination, as determined using the method described by He et al. [47].

### Gap Detection Threshold (GDT) and Speech Perception Outcome Assessments

In a subgroup of 10 children with ANSD included in Experiment 2, within-channel GDTs were assessed using electrophysiological measures of the electrically evoked auditory change complex, elicited by temporal gaps embedded in 800-ms trains of electrical pulses delivered at a pulse rate of 900 pulses per second (pps) and presented at the C level to a mid-array CI electrode (i.e., electrode 12). These results are referred to as objective GDTs in this study. In these 10 participants, aided speech perception in quiet was also evaluated using the Phonetically Balanced Kindergarten (PBK) word test, following age-appropriate clinical procedures. In a subgroup of 15 adult participants, within-channel behavioral GDTs were measured at two electrode locations that differed in AN neural synchrony, as indicated by their PLV values. The stimulus was a 500-ms train of electrical pulses with a pulse rate of 900 pps presented at the C level. GDTs were obtained using a three-alternative forced-choice paradigm with a three-down, one-up adaptive procedure. This within-subject design reduces the influence of interindividual differences in cognitive function or baseline temporal resolution, thereby enabling a clearer interpretation of the relationships between behavioral GDTs and peripheral measures of IPL, IPLVAR, and PLV metrics. For adult CI users, aided AzBio sentence scores were obtained in quiet and in a 10-talker babble noise at signal-to-noise ratios (SNRs) of +10 and +5 dB, following standard clinical practice. Details of these assessments are provided in our previous studies [17, 43].

### Data Analyses

#### Calculation of the IPL and the IPLVAR

**Fig. 1:**
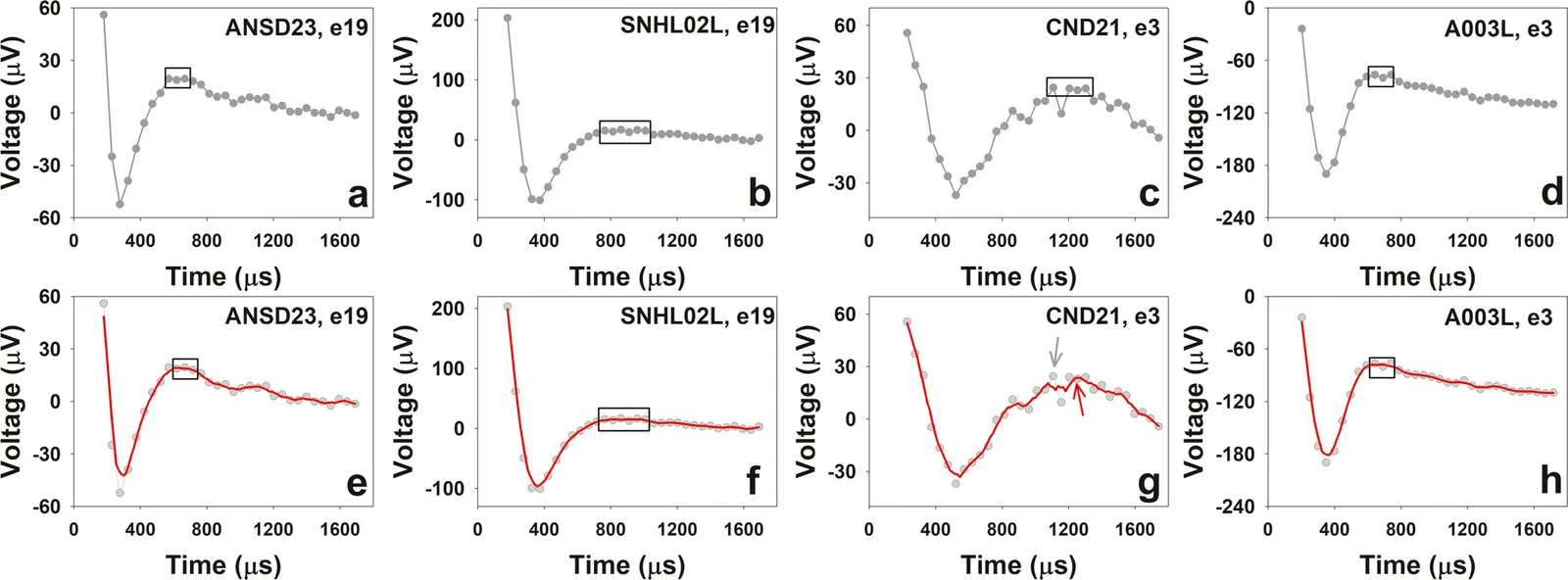
Examples of averaged eCAP responses (upper panels) and their corresponding smoothed waveforms (red lines in lower panels) recorded in one patient from each patient group with the multipoint peak issue. The time windows where multiple sampled points showed the same values are indicated using rectangles. Gray and red arrows in panel (**g**) denote the P2 peak locations identified from the unsmoothed and smoothed waveforms, respectively, without any mitigation applied to address the multipoint peak issue

In our datasets, both single-sweep and averaged eCAP waveforms frequently exhibited multiple sampled points within the designated latency windows for the N1 and P2 peaks that had nearly identical minimum or maximum voltage values. This issue is illustrated in examples shown in panels (a)–(d) of Fig. 1. The time windows containing these points are highlighted in the bolded black rectangles. Closer inspection revealed that the voltage differences among these points were less than 0.5 μV, substantially smaller than the estimated recording noise for Cochlear™ Nucleus® devices, which is approximately 2–5 μV [51–53]. However, the latency difference between these sampled points was 97.6 μs (i.e., two samples) or greater. Consequently, this “multipoint peak” issue introduced variability in the calculation of IPL and its IPLVAR, depending on which sampled point was selected to represent the peak latency.

Traditionally, N1 and P2 latencies have been determined by identifying the minimum and maximum voltage values within designated latency windows or sample points [54]. Using this approach, single N1 and P2 peaks are identified even when the voltage differences between neighboring samples are as small as 0.0001 μV (i.e., the smallest measurable voltage difference using the setup described in the current study), despite the fact that these small voltage differences are well below the noise floor of CI devices and therefore not clinically meaningful. As a result, the presence of “multipoint peak” issues in eCAP waveforms and their impact on latency estimation have not been well documented or systematically addressed. In an initial attempt to address this issue, we applied local smoothing techniques to the recorded responses; however, this approach did not fully resolve the problem. To better illustrate this, the red lines shown in panels (e)–(h) of Fig. 1 represent the smoothed waveforms corresponding to the eCAP responses displayed in the upper panels of the same figure. Smoothing was performed using a negative exponential smoother that employed polynomial regression with a first-degree polynomial and weights derived from a Gaussian density function. The sampling proportion was set to 0.1, indicating that each smoothed value was calculated based on the nearest 10% of the total data points. As shown in panels (e), (f), and (h), the smoothed waveforms exhibited a plateau within the same time window where the multipoint peak issue was observed in the recorded eCAP waveforms. This problem persisted even when different smoothing techniques and sampling proportions were used. Consequently, local smoothing techniques were not applied in the present study.

To mitigate this multipoint peak issue, we applied a peak-picking method that took this issue into consideration to a certain extent. Specifically, N1 and P2 latencies were identified within defined latency windows by first rounding all voltage values to the nearest integer (in μV) prior to peak selection. This rounding introduced an additional voltage fluctuation of less than 0.5 μV on average, which was more conservative than the lowest noise floor reported for Cochlear™ Nucleus® eCAP recordings (~ 2 μV) [51] and therefore served as a conservative estimation for the impact of the multipoint peak issue on study results. For traces exhibiting the multipoint peak issue, a linear average of the latencies of all peak points within the designated latency window for the N1 or P2 peak was calculated and used as the corresponding N1 or P2 latency. For traces without this issue, N1 and P2 peak latencies were determined using the traditional method described previously. The IPL was defined as the difference between these two peak latencies (i.e., P2 minus N1). The variability of the IPL (i.e., IPLVAR) was defined as the standard deviation of the corresponding IPL values of the individual eCAP single sweeps recorded at each electrode location in each adult participant. It was calculated using the equation in the form of

$$IPLVAR= \sqrt{\frac{\sum_{i=1}^{N}{\left(IPLi-IPLmean\right)}^{2}}{N-1}}$$

where IPLi represents the IPL measured for each individual sweeps, IPLmean indicates the mean IPL calculated across all individual eCAP sweeps, and N refers to the number of individual eCAP sweeps.

In Experiment 2, IPLs were calculated from eCAPs recorded in three pediatric patient groups to enable comparisons of IPLs across these groups and examine their associations with objective GDT and PBK scores in children with ANSD. For adult CI users, the IPL was calculated based on the averaged eCAP response across individual sweeps. Compared with other participant groups, children with CND exhibited prolonged N1 latencies [55]. Consequently, the N1 latency window for this group was defined as 150–600 μs. For all other participant groups, the N1 latency window was defined as 150–400 μs [50]. For all participant groups, the P2 was identified as the positive peak occurring between the N1 and 1000 μs.

#### Calculation of the PLV

The PLV, which quantifies trial-to-trial phase coherence across all eCAP sweeps, was computed using the method described by He et al. [17]. Briefly, the PLV was derived using Hanning Fast Fourier Transform (FFT) functions at six frequencies ranging from 788.2 to 4729.2 Hz, with a step size of 788.2 Hz, a pad ratio of 2, and a frame size of 26 samples. The lower bound (788.2 Hz) was set by the FFT frame duration of 1268.8 μs, which was determined by the IPL reported for CI users [50] with typical SNHL and the 48.8-μs sampling resolution of Cochlear™ Nucleus® devices. The upper bound (4729.2 Hz) was chosen based on FFT analyses of averaged eCAPs (over 400 sweeps) from adult CI users, which showed that the highest harmonic with at least one-quarter of the amplitude of the fundamental frequency occurred at 4482.6 Hz. Using the spectral resolution of 788.2 Hz determined by the frame size, this yielded an upper frequency of 4729.2 Hz for the PLV calculation. At each electrode location, a single PLV was obtained by averaging the PLVs calculated from six partially overlapping frames spanning the eCAP time window in human CI users. For additional methodological details, see He et al. [17].

#### Recording Noise Analysis

For each electrode tested in each participant in Experiment 3 (i.e., the 0 clinical unit recordings), the single-sweep eCAP recording noise frequency spectrum averaged across 400 trials was calculated using FFT. The percentages of recording noise power within and below the frequency range of the PLV analysis (i.e., 788.2 to 4729.2 Hz) were computed.

### Statistical Analyses

Linear mixed-effects models (LMMs) with patient group and electrode location as fixed effects were used to compare IPLs and IPLVARs at three electrode locations across the three pediatric participant groups. LMMs with electrode location as a fixed effect were used to compare IPLs and IPLVAR values across electrode locations for adult CI users. All LMMs used a correlated regression model with an unstructured correlation matrix to account for repeated observations per participant. The two ears of bilateral users, if both were tested, were treated as separate participants. Estimations were obtained using restricted maximum likelihood with Satterthwaite degrees of freedom. Bonferroni correction was used to adjust for multiple comparisons.

Based on the results of Shapiro-Wilk tests assessing data normality, either Spearman rank or Pearson correlation analyses were conducted (1) to examine how IPLs and IPLVARs were associated with PLVs in all four participant groups in Experiment 1; (2) to evaluate the associations between IPLs and objective GDTs and between IPLs and PBK scores, measured in 10 children with ANSD in Experiment 2; and (3) to assess the effects of age on the IPL and IPLVAR at different electrode locations in adult CI users. For these tests, Bonferroni correction was used to adjust for multiple comparisons as needed.

Multiple linear regressions (MLRs) with AzBio score as the outcome and either the IPL or the IPLVAR as the predictor were performed to evaluate the association between speech perception outcomes and each of these two parameters under each testing condition (quiet, + 10 dB, and + 5 dB SNR). Separate MLR models were constructed: One set included participant age as a covariate to control for its potential influence on speech perception and/or eCAP measurements, while the other set did not. The MLRs were performed in R v4.4.1 (R Core Team, 2024). All other statistical analyses for this study were performed using SPSS Statistics (v. 28.0.0) (IBM, Armonk, NY). Statistical significance was determined at the 95% confidence level (i.e., *p* < 0.05).

### Simulation of the Effects of Intertrial eCAP Jitter, Sampling Rate, and Noise Level on the IPL, IPLVAR, and PLV

Figure 2 of Schvartz-Leyzac et al. [21] illustrates how intertrial jitter in the single-sweep eCAP waveform can lead to smearing out of the average eCAP waveform and a lengthening of the IPL of the average waveform. However, they only considered two extreme cases in their schematic illustration to justify the use of the IPL as a measure of neural synchrony. Specifically, the case shown in their Figure 2 A has a very small amount of jitter such that the average waveform is very similar to the template single-sweep waveform. In comparison, the second case, shown in their Figure 2B has a much larger amount of jitter such that the N1 peaks of some sweeps overlap with the P2 peaks of other sweeps, which leads to the smearing of the average eCAP waveform. It is unclear how much of this smearing of the average eCAP waveform occurs in cases with intermediate amounts of jitter and thus how sensitive the IPL of the average waveform is to various degrees of neural synchrony. To systematically investigate this issue, we have extended and modified the illustration of Schvartz-Leyzac et al. [21] in the following ways.

First, Schvartz-Leyzac and colleagues implemented a horizontal shift in their jitter simulation, but this leads to an identical single-sweep IPL for each trial. In our simulations performed in MATLAB (version R2024a), we have implemented the jitter as a linear time scaling (i.e., temporal compression or expansion around the point t=0) of the template eCAP waveform, such that the single-sweep IPL varies directly as a function of the scaling factor. This scaling-based approach to applying temporal jitter also leads to the P2 latency having a larger standard deviation than then N1 latency, consistent with the results of our latency analysis for the dataset collected at the C level in postlingually deafened adult CI users.

**Fig. 2:**
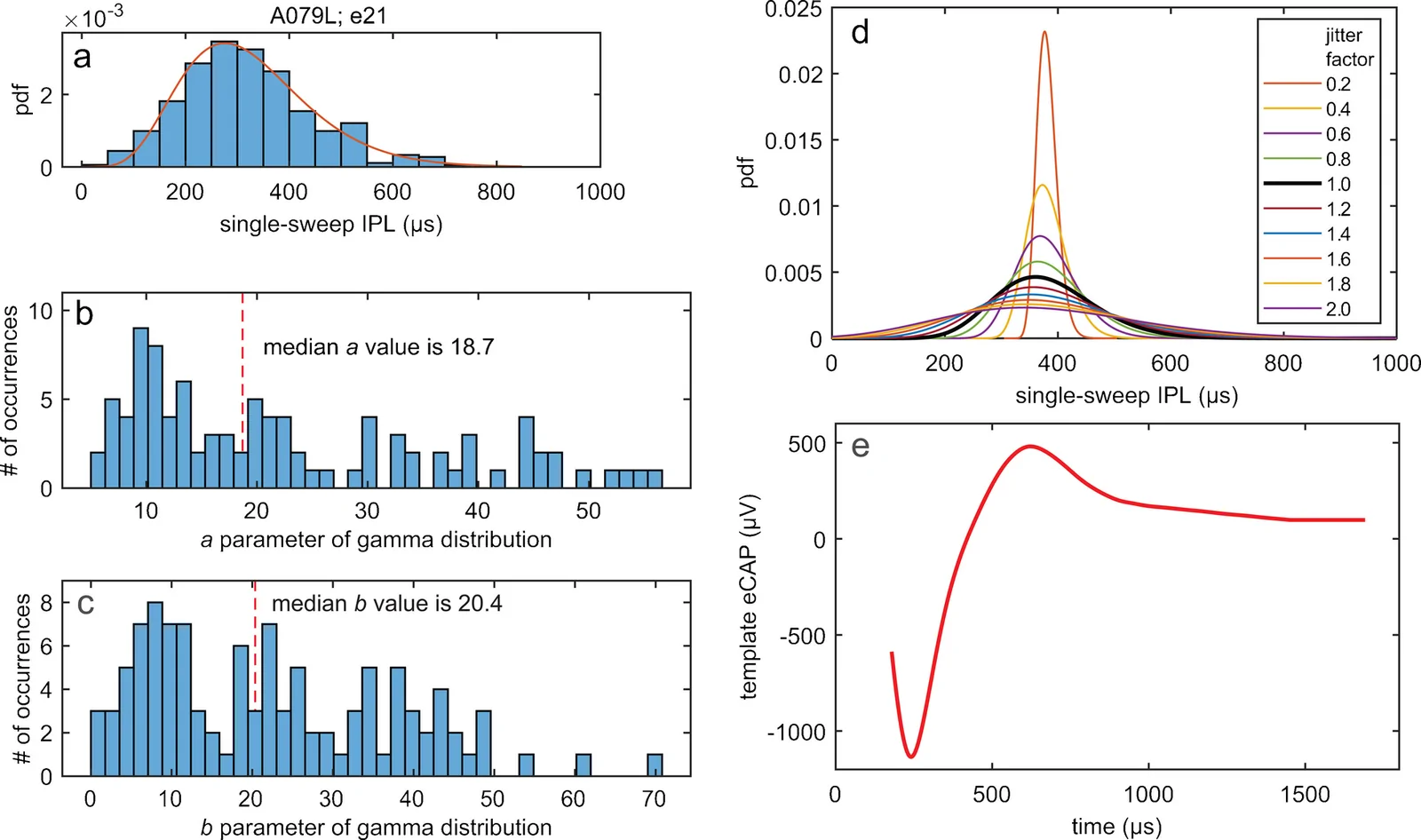
Methodology for simulation of intertrial jitter of the electrically evoked compound action potential (eCAP). **a** Histogram of single-sweep interpeak latencies (IPLs) measured at the maximum comfortable level (i.e., C level) for an example electrode. This distribution is well fit by a gamma distribution with the probability density function (pdf) shown by the red curve. **b**, **c** Histograms of the gamma distribution parameters *a* (shape) and *b* (scale), respectively, from fits to each electrode in each of 28 adult participants included in Experiments 1 and 2. **d** Distribution probability density functions (pdfs) of example gamma distributions that are used in the intertrial jitter simulations. The thick black curve shows the baseline distribution (*jitter factor* = 1.0) obtained from the median values of *a* and *b* from the histograms in panels (**b**) and (**c**). The other gamma distribution pdfs are obtained by scaling the baseline pdf around its mean by the value of the *jitter factor*. **e** Template eCAP waveform that is used for the intertrial jitter simulations, in this case for a sampling rate at 500 kHz

Second, we analyzed the IPL distributions for this dataset and found that they were generally well fit by a gamma distribution:

$$f\left(x\mid a,b\right)=\frac1{b^a\Gamma\left(a\right)}x^{a-1}e^{-x/b},\;for\;x>0\;and\;a,b>0,$$

where $\Gamma \left(\bullet \right)$ is the gamma function $\Gamma \left(x\right)={\int }_{0}^{\infty }{{e}^{-x}t}^{x-1} dt, x>0$, and a and b are the shape and scale parameters of the gamma distribution, respectively. Panel (a) of Fig. 2 shows the histogram of single-sweep IPLs for an example electrode, with the fitted gamma distribution probability density function (pdf) shown by the red curve. We used the function fitdist from the MATLAB Statistics and Machine Learning Toolbox to fit a gamma distribution pdf to the single-sweep IPL data measured at the C level for each electrode from each adult participant. Outliers were excluded using the MATLAB function isoutlier. Analysis of the goodness of the gamma-distribution fits is provided in Supplemental Digital Content 1. Panels (b) and (c) of Fig. 2 show the resulting histograms of the gamma distribution parameters *a* and *b*, respectively. The median values of the gamma distribution parameters were found to be *a* = 18.6 and *b* = 20.4.

Third, we designated the gamma distribution pdf with these median parameter values to be our baseline single-sweep IPL distribution for our simulations. Alternative gamma distributions with different amounts of jitter were then created by scaling the baseline pdf around its mean by a *jitter factor*, such that a jitter factor of 0.5 produces a single-sweep IPL standard deviation half that of the baseline pdf, while a jitter factor of 2 produces a standard deviation twice that of the baseline distribution. Jitter factors were tested from 0 to 2 in steps of 0.1, and ten simulations were run at each value of the jitter factor. Panel (d) of Fig. 2 shows pdfs of a subset of the gamma distributions that were used in the intertrial jitter simulations. For each run, 400 stimulus repetitions (trials) were implemented by generating a random number drawn from the appropriate gamma distribution using the function gamrnd from the MATLAB Statistics and Machine Learning Toolbox and applying the jittered linear scaling of the template eCAP waveform via the MATLAB function interp1. An analysis of the resulting IPL distributions obtained from the eCAP simulations provided in Supplemental Digital Content 2 indicates that the approach of choosing the median values of the gamma distribution parameters *a* and *b* and applying the jitter factor in the manner described above produces a range of simulated IPL distributions appropriate for exploring the effects of different degrees of spread around an average IPL value for the population.

Fourth, we created a template eCAP waveform for the simulations based on the average eCAP waveform from electrode 21 of participant A008R, as this electrode had the highest PLV value from the dataset collected at the C level in adult participants and a high SNR—see Fig. 2 of He et al. [17]. To investigate the effects of sampling rate on the synchrony metrics, a smoothed version of this waveform was generated by upsampling from 20.492 to 56, 100, and 500 kHz using piecewise cubic spline interpolation via the MATLAB function interp1. 20.492 and 56 kHz are the sampling rates used in Cochlear Corp. and Advanced Bionics eCAP measurement systems, respectively. The MED-EL eCAP system operates at a sampling rate of 1.2 MHz, but preliminary simulations showed negligible change in the synchrony metric estimates at sampling rates above 500 kHz. To reduce computation time, we therefore limited our analyses to a maximum sampling rate of 500 kHz. A linear horizontal scaling was then applied to the eCAP waveform at a given sampling rate via interp1 so that the waveform’s IPL matched the mean IPL from the gamma distribution pdfs shown in panel (d) of Fig. 2. The resulting template eCAP waveform at a sampling rate of 500 kHz is plotted in panel (e) of Fig. 2. In the simulations, jittered single-sweep waveforms were excluded as outliers if the N1 peak did not occur within the analysis window, consistent with the methodology used for the data analysis.

Fifth, in some simulations, simulated recording noise was added to each single-sweep eCAP waveform at SNRs of 18, 12, or 6 dB. This simulated recording noise had a Gaussian amplitude distribution and a magnitude-frequency spectrum that matched the average magnitude-frequency spectrum from the recording noise analysis shown in Fig. 10(a), with the spectrum extrapolated to the new Nyquist frequency (F_s_/2) for the simulations at sampling rates of 56, 100, and 500 kHz—see panel (a) of Fig. C in Supplemental Digital Content 5.

## Results

Results from Experiments 1 and 2 were presented in sequence first. Data related to the multipoint peak issue were then reported, followed by the results of Experiment 3, which evaluated the influence of recording noise. Finally, computational simulation results that demonstrated the effects of intertrial eCAP jitter, sampling rate, and recording noise on PLV, IPLVAR, and IPL metrics were presented.

In adult participants, the MLRs evaluating the associations between IPLs or IPLVARs and AzBio sentence scores, with and without controlling for the effect of age, yielded consistent results. Therefore, only the results of the MLRs that controlled for the effect of age, along with the additional variance explained by IPLVAR or IPL, which was calculated by subtracting the *R*^2^ value of the baseline model (AzBio ~ age) from that of the model including IPLVAR or IPL, are reported here. Results of the MLRs without controlling for the effect of age are provided in Supplemental Digital Contents 3 and 4.

It should be noted that behavioral GDTs were measured at two electrode locations differing in the PLV in 15 adult participants. The aim of including these two locations was not to compare basal versus apical positions, but to examine within-subject differences in AN neural synchrony and their relationship to GDT. Accordingly, the specific cochlear position of each electrode was not the focus of this investigation.

### Experiment 1: Evaluations of the IPLVAR

#### Between- and Within-Group Comparisons

**Fig. 3:**
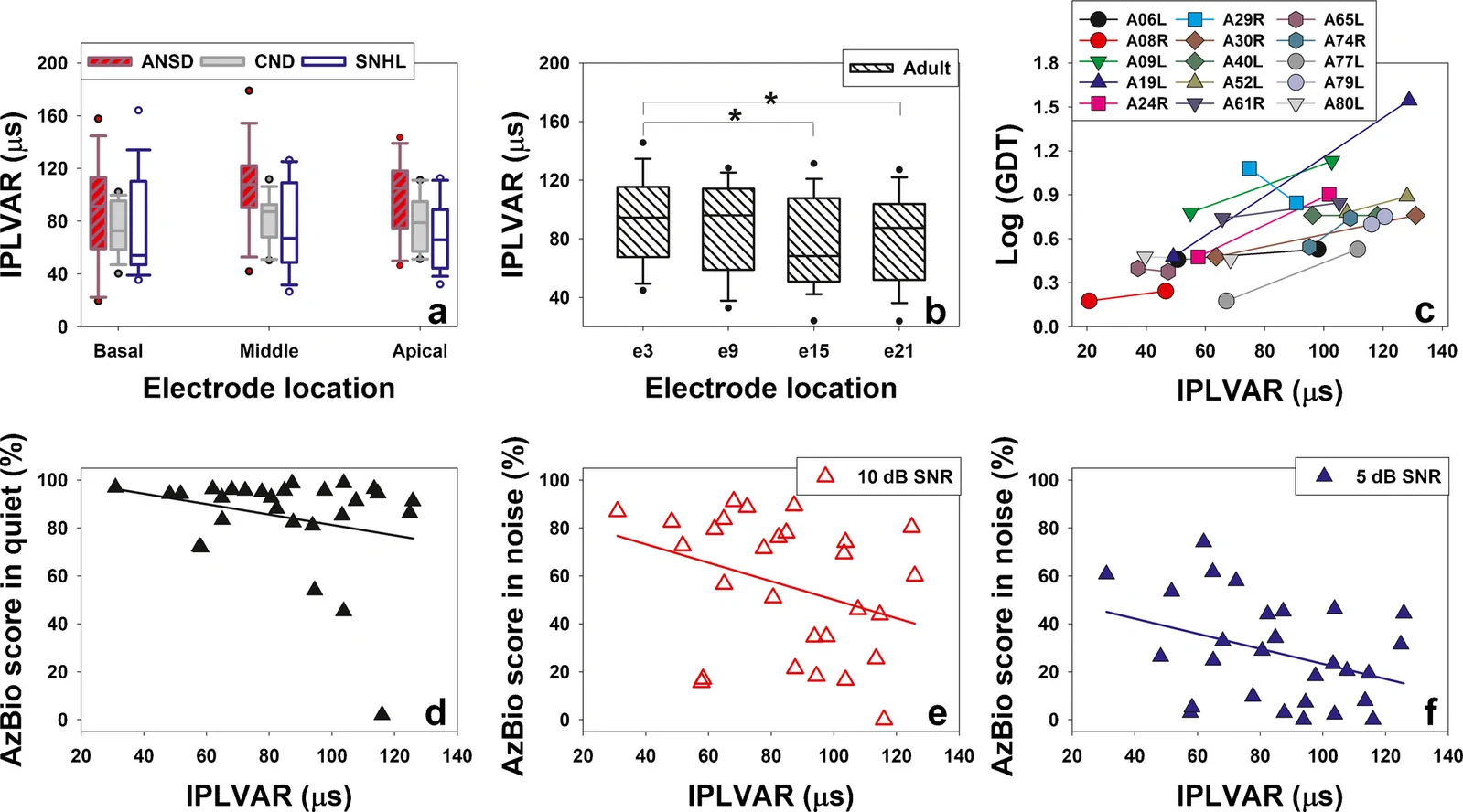
Group results of the intertrial variability of interpeak latency (IPLVAR) measurements. **a** Box plots of IPLVARs measured across all electrode locations in the three pediatric participant groups. **b** Box plots of IPLVARs measured at four electrode locations in adult participants. **c** IPLVAR values and psychophysical gap detection thresholds (GDTs) measured at two electrode locations in each of the 15 adult participants, with lines connecting data obtained from the same ear. **d**–**f** Scatter plots illustrating the associations between IPLVAR and AzBio sentence scores measured in quiet and in noise at signal-to-noise ratios (SNRs) of +10 dB and +5 dB, respectively. In panels (**a**) and (**b**), boxes represent the interquartile range (IQR; 25th–75th percentiles), horizontal bars within boxes indicate the median, and whiskers extend to 1.5 IQR. Dots denote the 5th and 95th percentiles. In panel (**b**), asterisks indicate statistically significant differences. In panel (**c**), each symbol represents data measured at one electrode location in one participant. In panels (**d**)–(**f**), each symbol represents data from an individual participant, and solid lines indicate the results of linear regression analyses

Panel (a) of Fig. 3 shows IPLVARs measured at three electrode locations in the three pediatric participant groups. Visual inspection suggested that children with ANSD tended to have larger IPLVARs than both children with CND and children with typical SNHL at all three electrode locations. Consistent with these observations, LMM analyses revealed a significant main effect of participant group (*F*_(2, 41.78)_ = 4.750, *p* = 0.014), but no significant effects of electrode location (*F*_(2, 39.99)_ = 0.819, *p* = 0.448) or the interaction between group and electrode location (*F*_(4, 39.63)_ = 0.536, *p* = 0.710). Post hoc comparisons showed that IPLVARs in children with ANSD were significantly greater than those in children with CND (*p* = 0.014). However, no significant differences were found between children with ANSD and those with SNHL (*p* = 0.131) or between children with CND and those with SNHL (*p* = 1.000).

Panel (b) of Fig. 3 shows IPLVARs measured at four electrode locations in adult participants. These results display an overall decreasing trend in the IPLVAR as the testing electrode moved to more apical electrode locations. Consistent with this observation, the results of a LMM revealed a significant effect of electrode location on the IPLVAR (*F*_(3, 23.08)_ = 3.939, *p* = 0.021). Post hoc analysis revealed that IPLVAR results measured at electrode 3 were significantly larger than those measured at electrodes 15 (*p* = 0.002) and 21 (*p* = 0.042). There was no significant difference in the IPLVAR between any other electrode location pairs (*p* > 0.05).

#### Associations with GDT and Speech Perception Scores

The association between psychophysical GDTs and IPLVAR values is shown in panel (c) of Fig. 3. All except for four participants (A029, A040, A065, and A080) showed lower GDTs at the electrode location with smaller IPLVAR values. The results measured in the right ear of A029 and in the left ears of A065 and A080 showed an opposite pattern. For participant A040, the same GDTs were measured at two electrode locations despite differing IPLVAR values; therefore, the data measured in A040 were excluded from the subsequent LMM analysis. An LMM revealed a significant relationship between the IPLVAR and the logarithmic transformation of GDT (logGDT) (*t*_(24.01_) = 3.990, *p* < 0.001). The intraclass correlation coefficient (ICC) was 0.58 and reached statistical significance (*p* = 0.029), indicating a significant within-participant association between the logGDT values. Panels (d)–(f) of Fig. 3 show the association between the numerically averaged IPLVAR across four electrode locations and AzBio sentence scores measured in different listening conditions. These results demonstrate the lack of association between AzBio sentence scores measured in quiet and the IPLVAR. AzBio sentence scores measured in the two noise conditions tended to be lower for larger IPLVAR values. The MLRs after controlling for the effect of age showed nonsignificant associations between IPLVARs and AzBio sentence scores measured in quiet or in noise at a SNR of 10 dB. The association between these two variables was statistically significant for the 5 dB SNR, with larger IPLVARs associated with lower AzBio sentence scores. Details of these results are listed in Table 3. Results of the MLR without controlling for the effect of age yielded consistent findings and are provided in Table A of Supplemental Digital Content 3.

**Table 3:** Results of multiple linear regressions (MLRs) for assessing the associations between AzBio sentence scores measured in different listening conditions and the interpeak latency variability (IPLVAR) results quantified in different ways after controlling the age effect

IPLVAR calculation method	Listening condition	Predictor	Unstandardized *β *(*SE*)	*t*	*p*	*R*^2^	Additional variance explained by the IPLVAR
IPLVAR	Quiet	IPLVAR	−0.233 (0.157)	1.485	0.150	0.083	8.08%
		Age	0.094 (0.269)	0.351	0.729		
	Noise, SNR + 10 dB	IPLVAR	−0.387 (0.203)	1.903	0.069	0.173	11.98%
		Age	−0.397 (0.348)	1.142	0.264		
	Noise, SNR + 5 dB	IPLVAR	−0.316 (0.144)	2.205	0.037^*^	0.294	13.72%
		Age	−0.542 (0.246)	2.207	0.037^*^		
IPLVAR_min_	Quiet	IPLVAR	−0.229 (0.123)	1.855	0.075	0.123	12.07%
		Age	0.119 (0.264)	0.450	0.657		
	Noise, SNR + 10 dB	IPLVAR	−0.317 (0.162)	1.954	0.062	0.179	12.55%
		Age	−0.371 (0.348)	1.065	0.297		
	Noise, SNR + 5 dB	IPLVAR	−0.253 (0.115)	2.198	0.037^*^	0.294	13.64%
		Age	−0.522 (0.247)	2.116	0.045^*^		
IPLVAR_max_	Quiet	IPLVAR	−0.167 (0.131)	1.268	0.216	0.063	6.03%
		Age	0.083 (0.272)	0.304	0.763		
	Noise, SNR + 10 dB	IPLVAR	−0.284 (0.171)	1.664	0.109	0.148	9.44%
		Age	−0.416 (0.353)	1.179	0.249		
	Noise, SNR + 5 dB	IPLVAR	−0.262 (0.119)	2.204	0.037^*^	0.294	13.71%
		Age	−0.555 (0.245)	2.261	0.033^*^		

*SNR* signal to noise ratio; *SE* standard error; *statistically significant

### Associations with the PLV

**Fig. 4:**
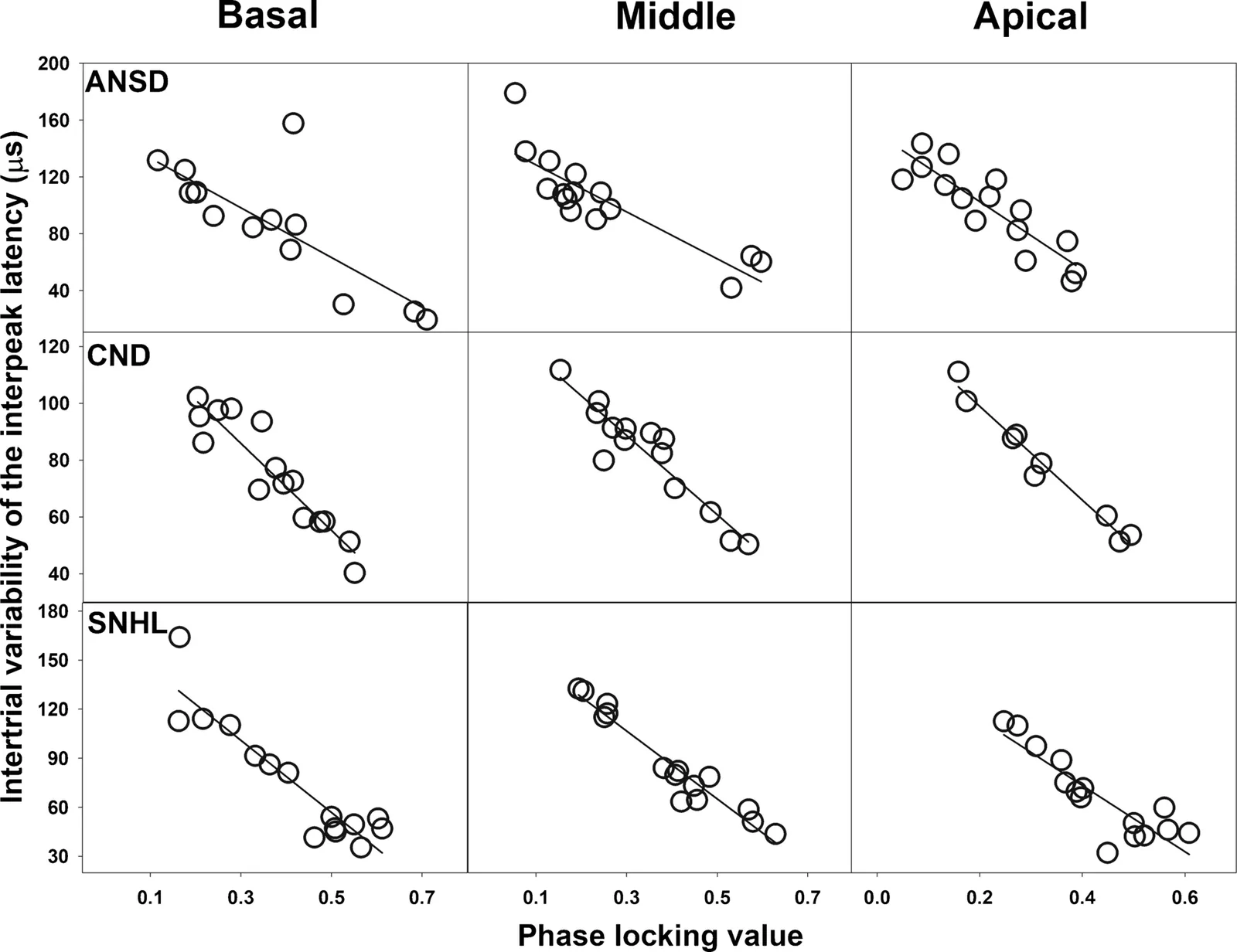
Associations between the phase-locking value (PLV) and the intertrial variability of the interpeak latency (IPLVAR) are shown for the three pediatric participant groups: children with auditory neuropathy spectrum disorder (ANSD), children with cochlear nerve deficiency (CND), and children with typical sensorineural hearing loss (SNHL). Each row represents data obtained from one participant group, and each panel corresponds to a specific electrode location. Individual symbols denote data from individual participants, and solid lines represent the results of linear regression analyses

**Fig. 5:**
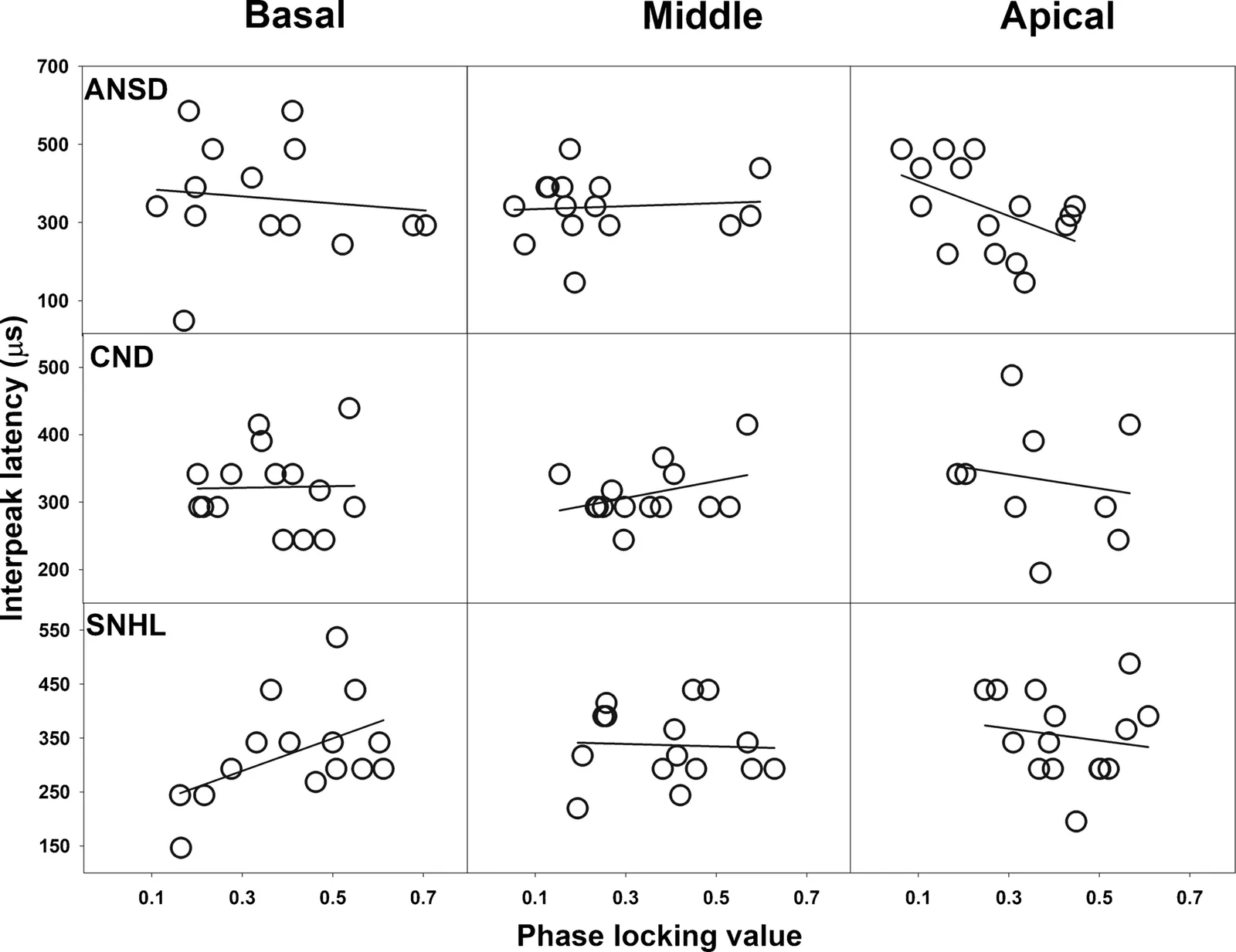
Associations between the phase-locking value (PLV) and the interpeak latency (IPL) are shown for the three pediatric participant groups: children with auditory neuropathy spectrum disorder (ANSD), children with cochlear nerve deficiency (CND), and children with typical sensorineural hearing loss (SNHL). Each row represents data obtained from one participant group, and each panel corresponds to a specific electrode location. Individual symbols denote data from individual participants, and solid lines represent the results of linear regression analyses

**Fig. 6:**
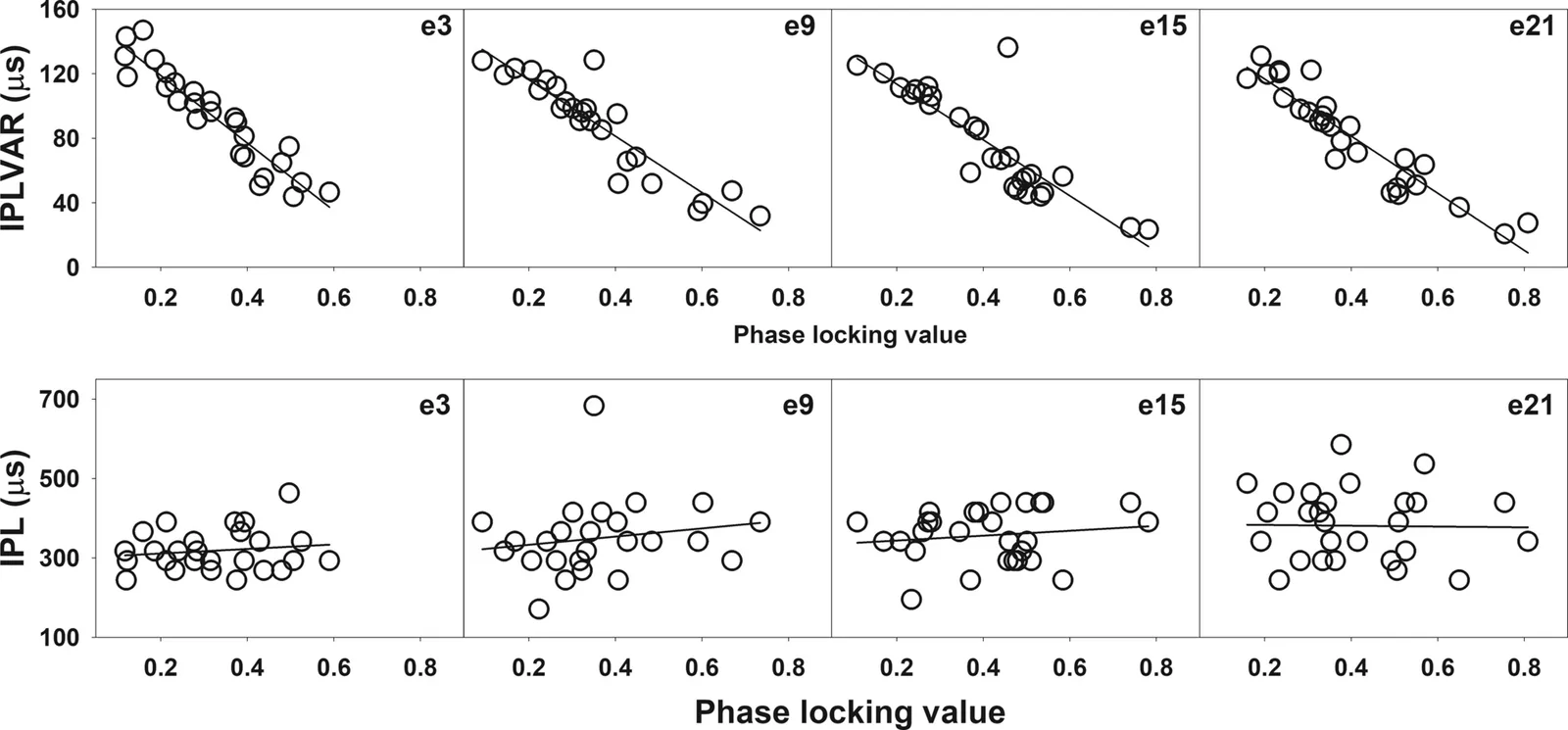
Associations between the phase-locking value (PLV) and the intertrial variability of the interpeak latency (IPLVAR) (upper panels), as well as between the PLV and the IPL (lower panels) for adult participants. Each panel shows the results measured at one electrode location, and each symbol represents data from an individual participant. Solid lines indicate the results of linear regression analyses

Figures 4 and 5 illustrate the associations between the PLV and the IPLVAR and between the PLV and the IPL, respectively, at each electrode location tested in the three pediatric participant groups in Experiment 1. Results obtained from adult CI users are shown in Fig. 6. Visual inspection revealed no consistent association between the PLV and the IPL across electrode locations in any participant group. In contrast, larger IPLVARs tended to be associated with smaller PLV values across all groups. These visual observations were confirmed by Pearson or Spearman rank correlation analyses. Specifically, Shapiro-Wilk tests indicated that PLVs measured at electrode 12 in children with ANSD (*W*_(9)_ = 0.685, *p* < 0.001), IPLVARs measured at electrode 3 in children with SNHL (*W*_(9)_ = 0.806, *p* = 0.024), as well as IPLs measured at electrode 3 (*W*_(9)_ = 0.794, *p* = 0.017), and IPLVARs measured at electrode 15 (*W*_(9)_ = 0.781, *p* = 0.013) in adult CI users did not follow a normal distribution. As a result, Spearman rank correlation tests were used to assess the associations between the PLV and the IPLVAR and/or between the PLV and the IPL in these cases. All other variables were normally distributed (*p* > 0.05), and the associations between these variables were assessed using Pearson correlation tests. These analyses revealed significant correlations between the IPL and the IPLVAR across all four groups and at all electrode locations. In contrast, no significant correlations were found between the IPL and the PLV in any participant group at any electrode location. Details of these correlation analyses are provided in Table 4.

**Table 4:** Results of Pearson and Spearman rank correlation analyses showing the associations between the phase-locking value (PLV) and the intertrial variability of the interpeak latency (IPLVAR), as well as between the PLV and the interpeak latency (IPL), at each electrode location across the four participant groups

Group	Electrode location	Variable assessed with the PLV			
		IPLVAR		IPL	
		Correlation coefficient	*p* value	Correlation coefficient	*p* value
ANSD	e3	*r*_(12)_ = −0.797	< 0.001^*^	*r*_(12)_ = −0.116	0.693
	e12	*ρ*_(13)_ = −0.825	< 0.001^*^	*ρ*_(13)_ = −0.056	0.842
	e21	*r*_(13)_ = −0.879	< 0.001^*^	*r*_(13)_ = −0.487	0.066
CND	Basal	*r*_(13)_ = −0.930	< 0.001^*^	*r*_(13)_ = 0.023	0.936
	Middle	*r*_(12)_ = −0.936	< 0.001^*^	*r*_(12)_ = 0.365	0.199
	Apical	*r*_(7)_ = −0.984	< 0.001^*^	*r*_(7)_ = −0.163	0.675
SNHL	e3	*ρ*_(13)_ = −0.839	< 0.001^*^	*r*_(13)_ = 0.495	0.061
	e12	*r*_(13)_ = −0.968	< 0.001^*^	*r*_(13)_ = −0.046	0.871
	e21	*r*_(13)_ = −0.877	< 0.001^*^	*r*_(13)_ = −0.156	0.579
Adult	e3	*r*_(24)_ = −0.941	< 0.001^*^	*ρ*_(24)_ = 0.147	0.474
	e9	*r*_(23)_ = −0.916	< 0.001^*^	*r*_(23)_ = 0.174	0.405
	e15	*ρ*_(26)_ = −0.882	< 0.001^*^	*r*_(26)_ = 0.146	0.458
	e21	*r*_(26)_ = −0.938	< 0.001^*^	*r*_(26)_ = 0.074	0.709

*ANSD* auditory neuropathy spectrum disorder; *CND* cochlear nerve deficiency; *SNHL* sensorineural hearing loss; *statistically significant

### The Effect of Age on the IPL and IPLVAR

**Fig. 7:**
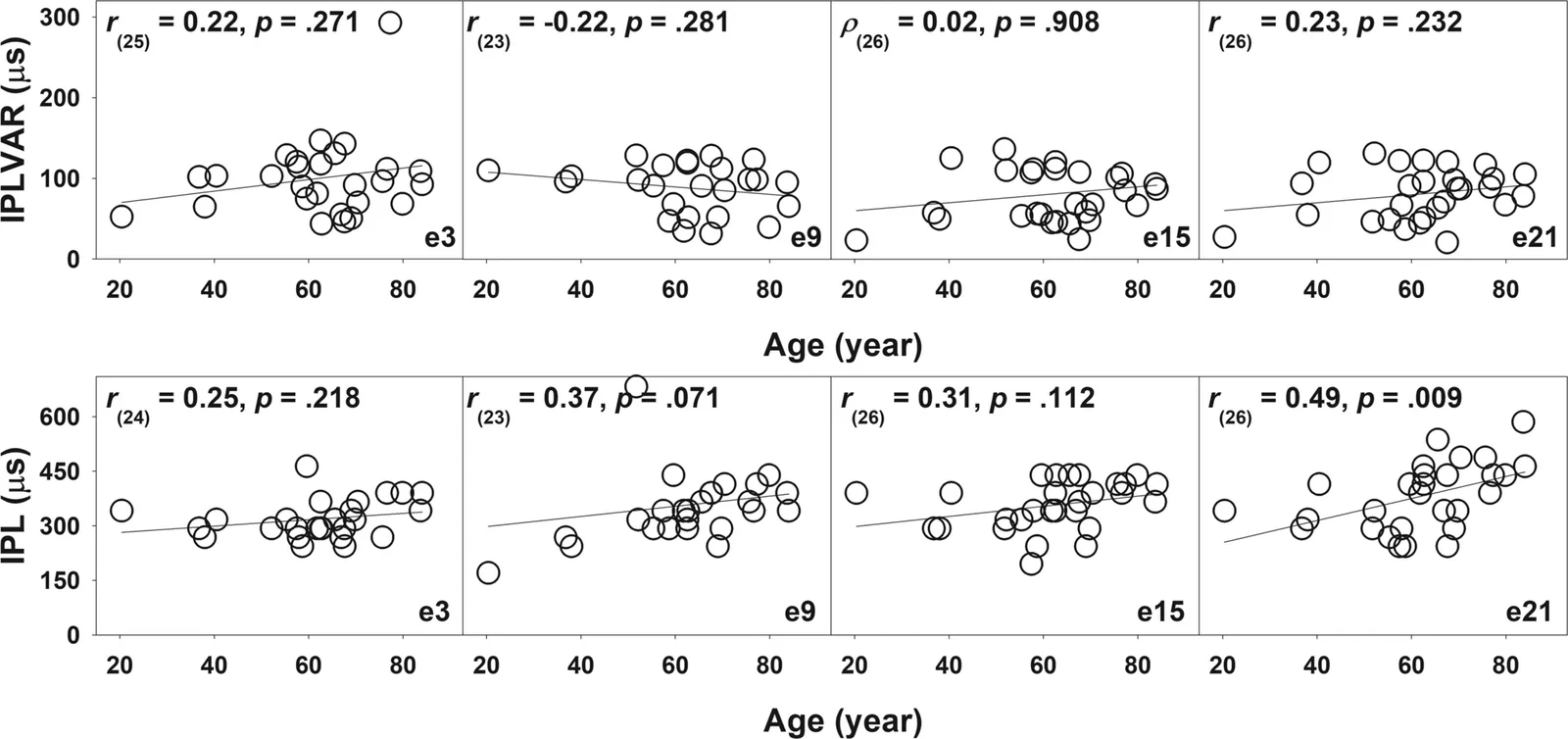
The association between age and the intertrial variability of the interpeak latency (IPLVAR) and between age and the interpeak latency (IPL) for adult participants. Upper and lower panels show the results of the IPLVAR and the IPL, respectively. Each panel represents the results measured at one electrode location. Each symbol indicates the result measured in one participant. Solid lines represent the results of linear regression. The results of Pearson and Spearman rank correlation tests without Bonferroni correction are indicated in each upper and lower panel, respectively

The upper and lower panels of Fig. 7 show IPLVARs and IPLs measured at different electrode locations in adult participants, plotted as a function of age, respectively. Shapiro-Wilk tests indicated that IPLVARs measured at the default electrode 15 did not follow a normal distribution (*W*_(23)_ = 0.911, *p* = 0.043), whereas all other variables were normally distributed (*p* > 0.05). As a result, a Spearman rank correlation test was used to assess the association between age and the IPLVAR at electrode 15, while Pearson correlation tests were applied for all other assessments. Pearson correlation analyses with Bonferroni correction revealed a statistically significant positive correlation between age and the IPL only at the most apical electrode examined (e21), but not at any other electrode locations, using a Bonferroni-corrected α level of 0.0125. In contrast, Spearman rank correlation tests showed no significant correlation between the IPLVAR and age at any electrode location.

### Experiment 2: Evaluations of the IPL

#### Between- and Within-Group Comparisons

**Fig. 8:**
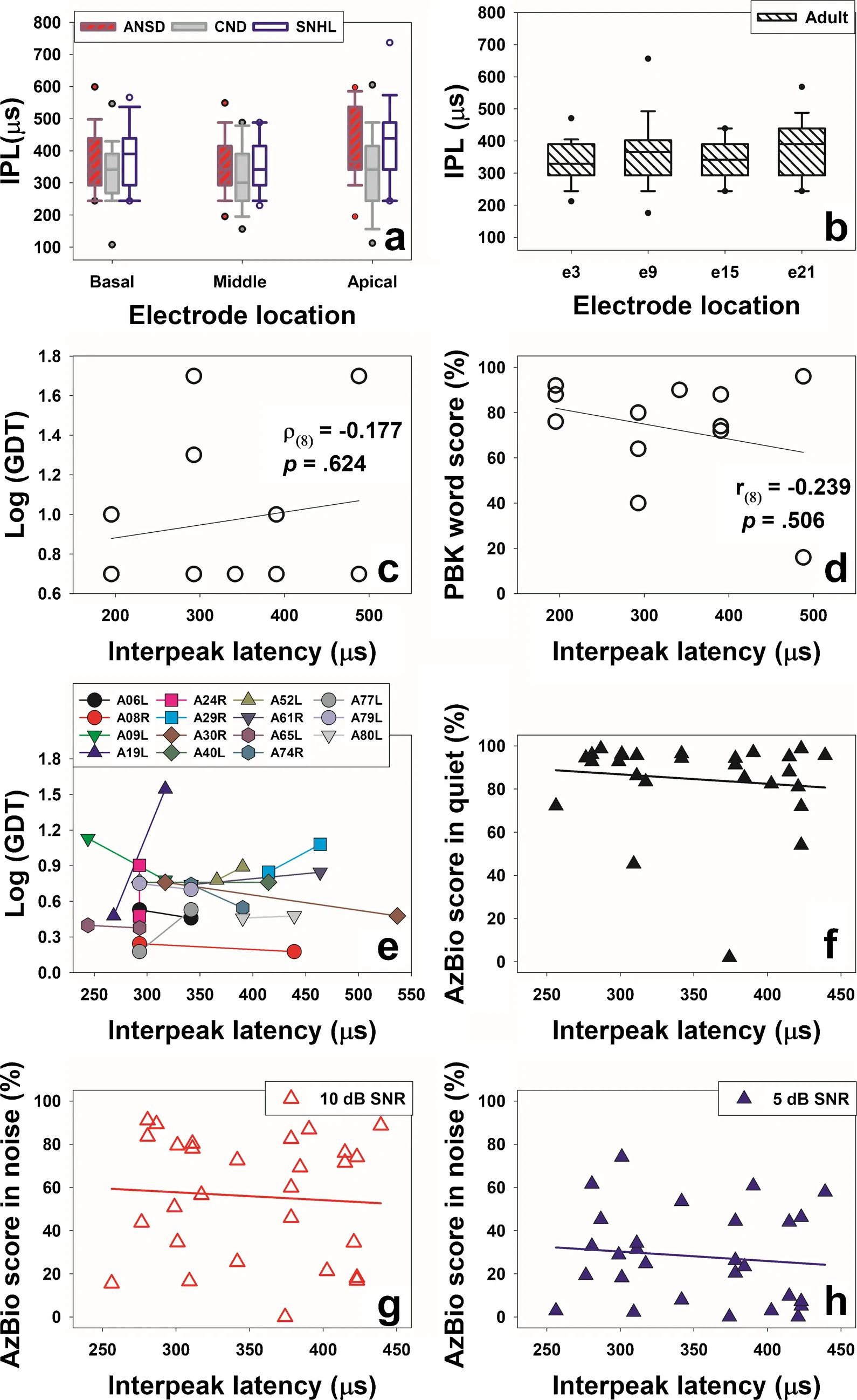
Interpeak latencies (IPLs) measured in different participant groups and their associations with gap detection thresholds (GDTs) and speech perception scores. **a** Box plots of IPLs measured across all electrode locations in the three pediatric participant groups. **b** IPLs measured at four electrode locations in adult participants. **c**, **d** Scatter plots showing the relationships between IPLs and objective GDTs and Phonetically Balanced Kindergarten (PBK) word scores for a subgroup of 10 children with auditory neuropathy spectrum disorder (ANSD), respectively. Results of correlation analyses are shown in each panel. **e** IPLs and behavioral GDTs measured at two electrode locations in each of 15 adult participants; lines connect data from the two locations tested in the same ear. **f**–**h** Scatter plots illustrating the associations between IPL and AzBio sentence scores measured in quiet and in two noise conditions in adult participants; each symbol represents one participant, and solid lines indicate linear regression results. In panels (**a**) and (**b**), boxes represent the interquartile range (IQR; 25th–75th percentiles), horizontal bars within boxes indicate the median, and whiskers extend to 1.5 IQR; dots denote the 5th and 95th percentiles. In panels (**c**–**h**), each symbol represents data from an individual participant

IPLs measured at three electrode locations in the three pediatric participant groups are shown in panel (a) of Fig. 8. Differences between patient groups and electrode locations were investigated using LMMs. Significant main effects were observed for participant group (*F*_(2, 84.43)_ = 7.189, *p* = 0.001) and electrode location (*F*_(2, 85.05)_ = 5.168, *p* = 0.008). The interaction between participant group and electrode location did not reach statistical significance (*F*_(4, 85.03)_ = 0.370, *p* = 0.829). Post hoc comparisons indicated that IPLs in children with CND were significantly shorter than those in children with ANSD (*p* = 0.009) and typical SNHL (*p* = 0.003). Across pediatric groups, IPLs were significantly shorter at the middle-array electrode than at the apical electrode location (*p* = 0.006). No other electrode pairs showed significant differences in the IPL (*p* > 0.05).

Panel (b) of Fig. 8 shows IPLs measured at four electrode locations in adult participants. These results do not suggest any systematic differences in IPLs across electrode locations. Consistent with this observation, results from a LMM revealed no significant effect of electrode location on IPL (*F*_(3, 23.24)_ = 2.698, *p* = 0.069).

### Associations with GDT and Speech Perception Scores

The associations between objective GDTs and IPLs measured at electrode 12 in a subgroup of 10 children with ANSD are shown in panel (c) of Fig. 8. Panel (d) shows PBK word scores measured in the same group of patients plotted against the IPL. These results indicated no association between IPLs and either of the two auditory perception outcomes. Shapiro-Wilk tests showed that only objective GDTs deviated from a normal distribution (*W*_(10)_ = 0.770, *p* = 0.006). Consequently, Spearman rank correlation and Pearson correlation analyses were used to evaluate the associations between IPLs and GDTs and between IPLs and PBK scores, respectively. These analyses revealed no significant correlations for both variable pairs. The results of the correlation tests are shown in each panel.

Panel (e) of Fig. 8 shows behavioral GDTs measured at two electrode locations per test ear in a subgroup of 15 adult participants as a function of the IPL. These results provide no evidence for a consistent trend in the association between the IPL and GDT across participants. Specifically, the two CI electrode locations tested in the right ear of A024 showed equal IPLs but different GDTs. In the left ear of A040, the same GDTs were measured for the two electrode locations with an IPL difference of 122 µs. For the rest of the participants, while smaller GDTs were observed at the electrode locations with shorter IPLs in six participants, results measured in the other seven participants showed an opposite pattern. An LMM excluding the data from A040 revealed no significant relationship between the IPL and the logGDT (*t*_(22.57_) = 0.585, *p* = 0.564). The ICC was 0.049 and did not reach statistical significance (*p* = 0.868), indicating that there was no significant within-participant association between the logGDT values.

Panels (f)–(h) of Fig. 8 show the associations between the numerically averaged IPL across four electrode locations and AzBio sentence scores measured in quiet and in noise in 28 adult participants, respectively. These data provide no evidence for an association between the IPL and AzBio sentence scores measured in any listening conditions. This observation was supported by the results of MLRs after controlling for the effect of participant age. Details of these results are listed in Table 5. Results of the MLR without controlling for the effect of age yielded consistent findings and are provided in Table B of Supplemental Digital Content 4.

**Table 5:** Results of multiple linear regressions (MLRs) for assessing the associations between AzBio sentence scores measured in different listening conditions and the interpeak latency (IPL) results quantified in different ways after controlling the age effect. Unstandardized *β* are reported here

IPL calculation method	Listening condition	Predictor	*β *(*SE*)	*t*	*p*	*R*^2^	Additional variance explained by IPL
IPL	Quiet	IPL	−0.072 (0.082)	−0.887	0.384	0.033	3.04%
		Age	0.201 (0.314)	0.641	0.527		
	Noise, SNR + 10 dB	IPL	0.022 (0.110)	0.203	0.840	0.055	0.16%
		Age	−0.483 (0.423)	−1.141	0.265		
	Noise, SNR + 5 dB	IPL	0.041 (0.079)	0.515	0.611	0.166	0.88%
		Age	−0.653 (0.304)	−2.151	0.041^*^		
IPL_min_	Quiet	IPL	−0.058 (0.081)	−0.720	0.478	0.023	2.02%
		Age	0.176 (0.315)	0.557	0.582		
	Noise, SNR + 10 dB	IPL	0.034 (0.108)	0.317	0.754	0.057	0.38%
		Age	−0.505 (0.421)	−1.199	0.242		
	Noise, SNR + 5 dB	IPL	0.055 (0.077)	0.709	0.485	0.174	1.66%
		Age	−0.680 (0.302)	−2.255	0.033		
IPL_max_	Quiet	IPL	−0.083 (0.080)	−1.030	0.313	0.043	4.06%
		Age	0.220 (0.311)	0.705	0.487		
	Noise, SNR + 10 dB	IPL	0.009 (0.109)	0.084	0.934	0.054	0.03%
		Age	−0.458 (0.422)	−1.087	0.287		
	Noise, SNR + 5 dB	IPL	0.024 (0.078)	0.308	0.761	0.160	0.32%
		Age	−0.623 (0.304)	−2.051	0.051		
Schvartz-Leyzac et al. (2025) IPL-400 sweeps	Quiet	IPL	0.183 (0.258)	0.709	0.485	0.022	1.97%
		Age	0.023 (0.284)	0.081	0.936		
	Noise, SNR + 10 dB	IPL	0.095 (0.345)	0.276	0.785	0.056	0.29%
		Age	−0.465 (0.380)	−1.222	0.233		
	Noise, SNR + 5 dB	IPL	0.055 (0.249)	0.219	0.828	0.159	0.16%
		Age	−0.592 (0.274)	−2.156	0.041^*^		
Schvartz-Leyzac et al. (2025) IPL-50 sweeps	Quiet	IPL	0.391 (0.219)	1.784	0.087	0.115	11.26%
		Age	−0.114 (0.283)	−0.405	0.689		
	Noise, SNR + 10 dB	IPL	0.512 (0.291)	1.760	0.091	0.158	10.43%
		Age	−0.680 (0.376)	−1.811	0.082		
	Noise, SNR + 5 dB	IPL	0.287 (0.215)	1.334	0.194	0.213	5.60%
		Age	−0.712 (0.278)	−2.566	0.017^*^		

*SNR* signal to noise ratio; *SE* standard error; *statistically significant

### Multipoint Peak Issue

Using a voltage rounding threshold of 1 μV (i.e., the voltage values in μV were rounded to the nearest integer), the multipoint peak issue was observed in single eCAP sweeps measured in all participants in Experiment 1, even in cases where eCAP amplitude was 1000 µV or larger. The percentage of traces exhibiting the multipoint peak issue, regardless of whether it occurred for the N1 or P2 peak, ranged from 26.00 to 82.78% (mean 67.08%, SD 13.07%), from 30.56 to 80.73% (mean 61.03%, SD 11.04%), from 65.78 to 89.67% (mean 78.57%, SD 5.18%), and from 26.90 to 78.73% (mean 64.94%, SD 10.71%) in children with ANSD, children with SNHL, children with CND, and adult CI users, respectively. For the averaged eCAP response across 50–100 sweeps used in Experiment 2, the multipoint peak issue was observed in 20 out of 86 (23.26%), 15 out of 82 (18.29%), and 16 out of 93 (17.20%) eCAPs recorded in children with ANSD, children with SNHL, and children with CND, respectively. This issue was observed in 18 of the 107 averaged eCAPs (16.82%) derived from the first 50 sweeps recorded in adult CI users.

To better understand the impact of the multipoint peak issue on the study results and conclusions, the shortest IPL (IPL_min_) and the longest IPL (IPL_max_) were measured. IPL_min_ was defined as the difference between the shortest P2 latency and the longest N1 latency, whereas IPL_max_ was defined as the difference between the longest P2 latency and the shortest N1 latency. For traces without the multipoint peak issue, the same IPL value was used for both IPL_min_ and IPL_max_. The variabilities of IPL_min_ (IPLVAR_min_) and IPL_max_ (IPLVAR_max_) were also calculated for all participant groups. Their associations with GDTs and AzBio sentence scores were evaluated in adult CI users.

The difference between the IPLVAR_min_ and IPLVAR_max_ ranged between −56.12 μs and 65.78 μs (mean 6.62 μs, SD 22.90 μs), between −8.34 μs and 33.65 μs (mean 11.05 μs, SD 10.02 μs), between −28.37 μs and 58.32 μs (mean 11.69 μs, SD 16.56 μs), and between −28.51 μs and 53.99 μs (mean 11.20 μs, SD 14.91 μs) for children with ANSD, children with SNHL, children with CND, and adult CI users, respectively. The difference between the IPL_min_ and IPL_max_ ranged between 48.80 μs (one sample) and 146.40 μs (three samples) (mean 82.96 μs, SD 35.76 μs), between 48.80 μs (one sample) and 341.60 μs (seven samples) (mean 94.35 μs, SD 81.38 μs), between 48.80 μs (one sample) and 195.20 μs (four samples) (mean 91.50 μs, SD 46.72 μs), and between 48.80 μs (one sample) and 292.80 μs (six samples) (mean 81.33 μs, SD 57.98 μs) for children with ANSD, children with SNHL, children with CND, and adult CI users, respectively. These findings demonstrate that the multipoint peak issue introduces variability in both IPLVAR and IPL measurements across patient groups and can span a range of up to 121.90 μs and 341.60 μs (seven samples), respectively.

The association between the IPLVAR_min_ and GDT was similar to that observed for the IPLVAR. In contrast, for the IPLVAR_max_, the association between these two variables for the electrode pair tested in the left ear of participant A079 showed an opposite pattern relative to the associations observed for both the IPLVAR and the IPLVAR_min_. Neither the IPL_min_ nor the IPL_max_ exhibited a consistent trend in its associations with GDT, which is consistent with the results obtained for IPL. However, in three ears tested (A030R, A052L, and A079L), the association between GDT and IPL differed depending on how IPL was quantified, with the IPL_min_, the IPL_max_, and the IPL yielding inconsistent patterns.

Results of the MLRs assessing the associations between AzBio sentence scores across listening conditions and the two indicators quantified using different methods, after controlling for the effect of age, are presented in Tables 3 and 5. Results of the MLRs without age control are provided in Supplemental Digital Contents 3 and 4. Overall, the associations between AzBio sentence scores and IPLVARs were consistent across quantification methods, with a significant association observed for the noise condition with an SNR of 5 dB. No significant associations were found between AzBio sentence scores and IPLs across quantification methods. However, the effect of age on AzBio sentence scores was statistically significant only for the IPL_max_ results.

**Fig. 9:**
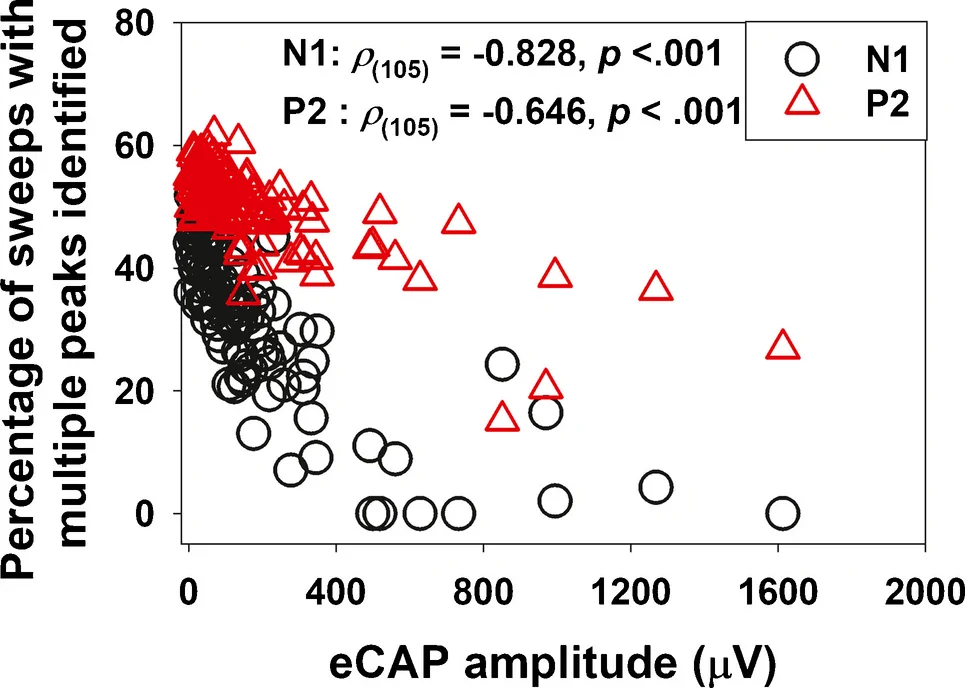
Percentages of traces showing multiple equal-value points for the N1 (black dots) and P2 (red triangles) peaks plotted against eCAP amplitude for all electrode locations in all adult participants. Each symbol represents data from one electrode location in one participant. Results of the Spearman rank correlation tests are shown in the panel

Figure 9 shows the percentages of eCAP traces in adult participants that exhibited the multipoint peak issue for the N1 and P2 peaks, plotted as a function of the average eCAP amplitude across sweeps. These data show that the percentage of traces with multiple peak points decreased as eCAP amplitude increased, suggesting an influence of recording noise. Spearman rank correlation tests revealed a significant negative correlation between the percentage and the average eCAP amplitude, confirming this visual observation.

### Experiment 3: Analysis of Recording Noise

**Fig. 10:**
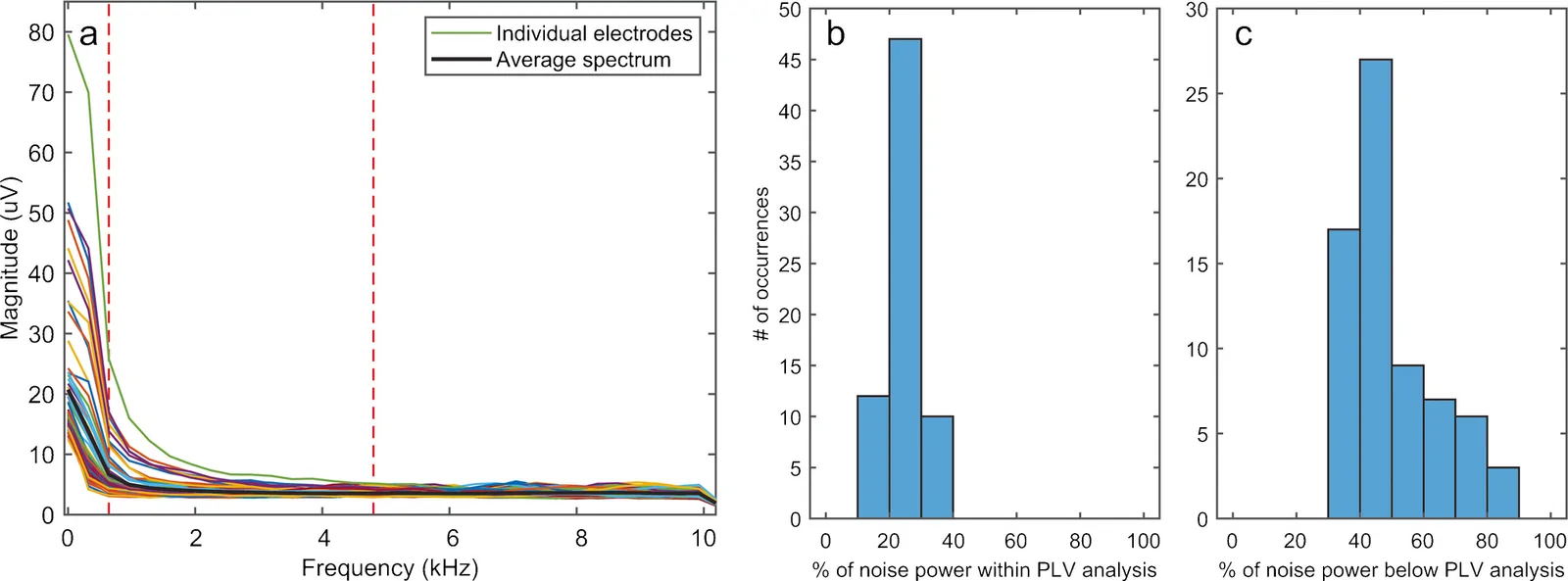
Recording noise analysis. **a** The individual (thin lines) and population average (thick black line) recording-noise spectrum for each of 69 electrodes across the 17 ears tested in 16 adult participants in Experiment 3. The vertical dashed red lines indicate the frequency range of the PLV analysis (0.789 to 4.73 kHz). Histograms for the percentage of the total recording noise power that is within the frequency range of the PLV analysis (**b**) and the percentage of power below that frequency range (**c**)

Panel (a) of Fig. 10 shows the individual (thin lines) and population average (thick black line) recording-noise spectrum for each of 69 electrodes across the 16 adult participants (17 ears) tested in Experiment 3. For the population average noise spectrum, only 22.5% of the noise power is within the frequency range of the PLV analysis (0.789 to 4.73 kHz), indicated by the red vertical dashed lines. In contrast, 58% of the population average recording noise power is below this frequency range. Panels (b) and (c) show histograms for the percentage of the recording noise power that is within the frequency range of the PLV analysis (panel b) and the percentage of power below that frequency range (panel c). Across all the electrodes in this dataset, the percentage of power within the PLV analysis frequency range extends from 10 to 40%, whereas the percentage of power below the PLV analysis range is between 30% and 90%. This indicates that electrodes with a greater amount of total recording noise power than the population average only have slightly more than average noise power within the PLV frequency range but have substantially more noise than the average at lower frequencies.

For these traces recorded at 0 clinical unit (0.4375 nC), IPLVARs and PLVs calculated from 400 individual eCAP sweeps ranged from 130.26 to 210.31 μs (mean 151.41 μs, SD 13.55 μs) and from 0.026 to 0.063 (mean 0.044, SD 0.009), respectively. The IPL measured from the averaged eCAP across sweeps ranged from 48.80 to 780.80 μs (mean 402.09 μs, SD 168.44 μs).

### Simulation of the Effects of Intertrial eCAP Jitter, Sampling Rate, and Noise Level

**Fig. 11:**
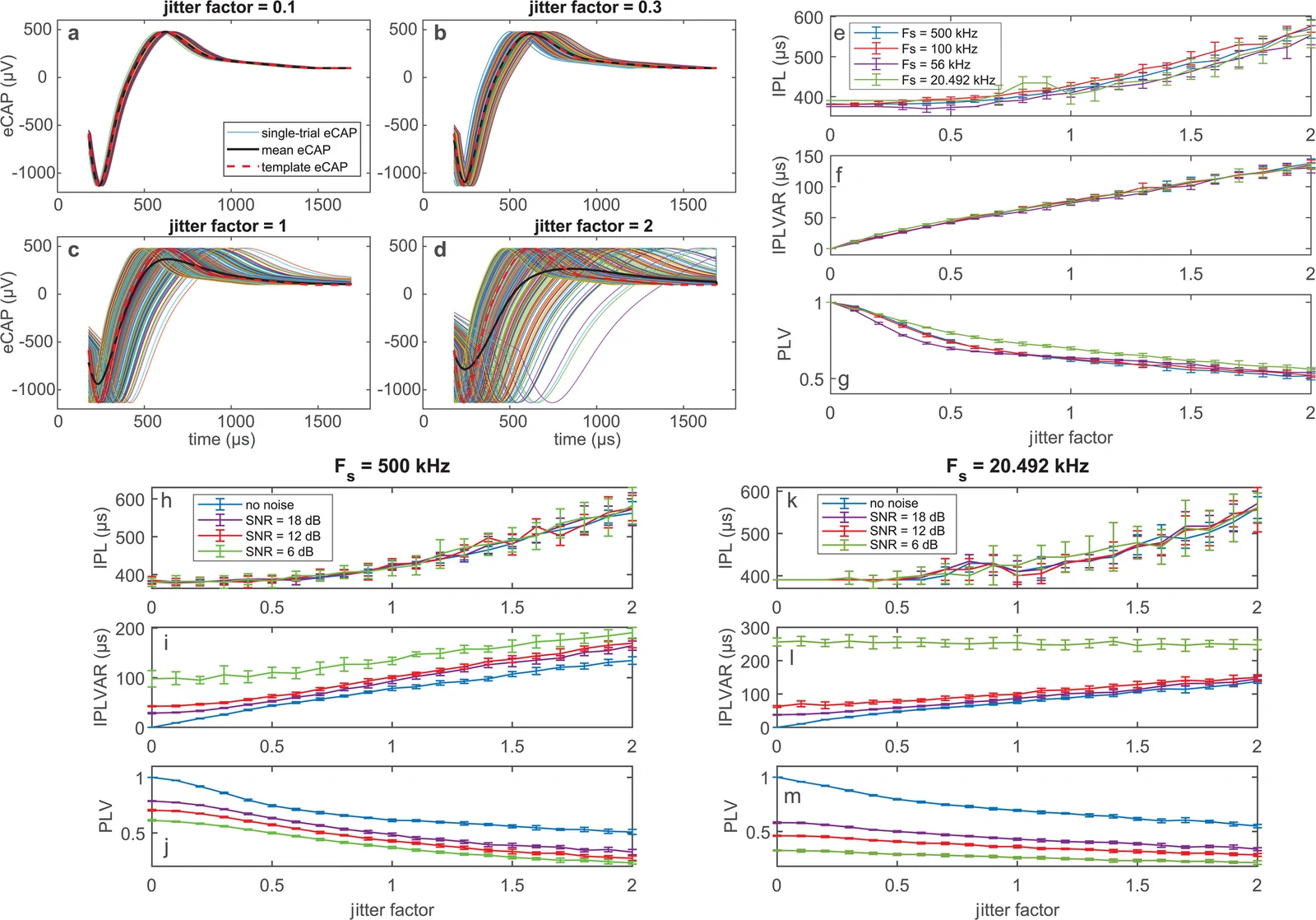
Jitter simulation results at different sampling rates and noise levels. **a**‒**d** Example results of the eCAP intertrial jitter simulations, for jitter factors of 0.1, 0.3, 1, and 2, respectively. These simulations were performed at a sampling rate of 500 kHz without simulated recording noise. Thin lines show the eCAP waveforms for single trials, thick black lines show the mean eCAP over all the trials, and thick dashed red lines show the template eCAP waveform. **e**‒**g** The estimated IPL of the mean eCAP waveform, the IPLVAR, and the PLV, respectively, as a function of the jitter factor for the different sampling rates indicated in the legend. **h**‒**m** The effects of simulated recording noise at the SNRs indicated in the figure legends on the synchrony metrics for sampling rates of 500 and 20.492 kHz, respectively. Each data point is the mean value from ten simulations run at that jitter factor, with error bars showing the standard deviation in the value over the ten repetitions

The results of the eCAP jitter simulations are shown in Fig. 11. Panels (a)–(d) provide example results of the eCAP intertrial jitter simulations with a sampling rate of 500 kHz without recording noise, for jitter factors of 0.1, 0.3, 1, and 2, respectively. It can be observed that increasing the jitter factor from 0.1 to 0.3 produces only minimal temporal smearing in the mean eCAP waveform (thick black lines) when compared to the template waveform (thick red dash lines), so the IPL is practically unchanged. When the jitter factor is increased to a value of 1 (i.e., to the baseline gamma distribution pdf that has a median amount of jitter) somewhat more temporal smearing occurs in the mean eCAP waveform and its IPL is elongated compared to the template waveform’s IPL. Increasing the jitter factor to 2 produces substantially more temporal smearing and a greatly lengthened IPL in the mean waveform.

Panels (e)–(g) of Fig. 11 show the mean values of the estimated IPL of the mean eCAP waveform, the IPLVAR over 400 trials, and the PLV over 400 trials, respectively, as a function of the jitter factor at sampling rates of 20.492, 56, 100, and 500 kHz, without recording noise. Error bars show the standard deviation in the value over ten simulation repetitions. At a sampling rate of 500 kHz, the IPL is insensitive to the jitter factor over the range of 0 to around 0.5 and then from 0.5 to 1 the IPL starts increasing and has a roughly linear relationship with the jitter factor for values between 1 and 2. However, the standard deviation of the estimates (indicated by the error bars) also grows in this region, indicating that it lacks somewhat in reliability even within this range of jitter factor values. As the sampling rate is reduced, the IPL estimates in panel (e) become more unreliable, as indicated by increased variability (larger error bars) and the emergence of nonmonotonic behavior in the relationship between the mean IPL estimate and jitter factor. In contrast, the IPLVAR estimates at the same sampling rate (500 kHz) in panel (f) exhibit a nearly linear relationship with the jitter factor over the entire range from 0 to 2, with only a moderate increase in the standard deviation of the estimates at larger jitter factor values. The PLV versus jitter factor relationship in panel (g) is not as linear as that for the IPLVAR, but it does not exhibit the same degree of flattening or nonmonotonicity as the IPL, remaining sensitive to changes in jitter factor over the full range. As the sampling rate is reduced, there is negligible change in the IPLVAR estimates and there are only modest changes in the shape of the PLV versus jitter factor curves, without any substantial increase in the size of the error bars. These simulation results suggest that the IPLVAR and PLV are more reliable measures of AN synchrony across a range of sampling rates than the IPL derived from the mean waveform.

For simulations at a sampling rate of 500 kHz, panel (h) of Fig. 11 shows that increasing levels of simulated recording noise also increase the unreliability of the IPL estimates, with larger error bars and nonmonotonic relationships emerging at the lower SNRs. In contrast, the IPLVAR estimates shown in panel (i) remain reliable at moderate SNRs, exhibiting only a small vertical shift in the curves and minimal change in the size of the error bars. However, at the lowest SNR of 6 dB, the IPLVAR versus jitter factor curve flattens out substantially, with increased error bars and some nonmonotonic behavior emerging. The PLV versus jitter factor curves shown in panel (j) show a similar vertical shift (downwards in this case) with negligible change in curve shape or error bar size, even at an SNR of 6 dB.

Panels (k)–(m) of Fig. 11 show that reducing the sampling rate to 20.492 kHz greatly exacerbates the effects of the SNR on the IPL estimates, as well as the impacts of recording noise on the IPLVAR estimates at an SNR of 6 dB. In contrast, the PLV versus jitter factor relationship at 20.492 kHz remains relatively robust to recording noise, exhibiting only slight curve flattening and a modest vertical shift as SNR decreases, without noticeable increase in error bar size. Intermediate effects of noise on these synchrony metrics were observed for simulations performed at 100 and 56 kHz—see panels (b)–(d) and (e)–(g), respectively, of Figure C in Supplemental Digital Content 5.

Overall, these simulation results indicate that the IPLVAR and PLV are more robust to sampling rate and moderate levels of recording noise than the IPL, with the IPLVAR becoming unreliable when the recording noise level is very high. To demonstrate that the results of these simulations are robust to the choice of IPL distribution type and the template eCAP waveform, additional simulations were run with a normal distribution instead of a gamma distribution (Supplemental Digital Content 6) and with four alternative eCAP templates (Supplemental Digital Content 7), all conducted at a sampling rate of 500 kHz without recording noise. The results of these additional simulations are qualitatively similar to those of the main simulations and demonstrate that, in some cases, the IPL versus jitter factor relationship can be nonmonotonic—not just shallow—for jitter factors between 0 and 1, even at a high sampling rate and in the absence of recording noise (see the curves for e15 and e9 of A008R in Supplemental Digital Content 7).

## Discussion

This study evaluated the IPLVAR and the IPL as potential indicators of neural synchrony in the AN in CI users. Both metrics require accurately identifying latencies of the N1 and P2 peaks of the eCAP. In this section, the results of each assessment were presented first, followed by two key factors affecting their accuracy. We then compared our findings with Schvartz-Leyzac et al. [21] and concluded with a discussion of the advantages and limitations of the IPLVAR and PLV.

### Experiment 1: The Intertrial Variability of the Interpeak Latency of the eCAP

One aim of this study was to test the hypothesis that reduced spike synchronization within and across AN fibers would lead to greater intertrial variability in the eCAP, as quantified by the IPLVAR. Overall, our results support the use of the IPLVAR as an indicator of neural synchrony in the AN. The key findings supporting this conclusion are summarized below.

#### Finding 1

Children with ANSD, a population characterized by poor neural synchrony, showed significantly larger IPLVARs than children with CND, who have fewer but otherwise healthy AN fibers. Among adult CI users, significantly larger IPLVARs were observed at the most basal electrode location compared with more apical locations. These patterns are consistent with histological findings in human temporal bone studies describing AN anatomy in these participant groups [35, 44–46].

#### Finding 2

For 11 of 15 adult participants, consistent correlations were observed between GDT and the IPLVAR: Lower GDTs occurred at electrode locations with smaller IPLVAR values. Additionally, the IPLVAR was significantly associated with AzBio sentence scores measured in noise at an SNR of 5 dB. These findings align with previous literature linking reduced neural synchrony in the AN to impairments in temporal resolution and speech perception in noise (e.g., [1]).

#### Finding 3

The robust correlation between the IPLVAR and the PLV across four participant groups indicates that both measures likely assess common underlying biological mechanisms that produce temporal jitter in the single-sweep eCAP waveform, as is simulated in Fig. 11. This interpretation is further supported by the similarity in their results, including the effects of electrode location and their associations with temporal resolution and speech perception outcomes, as reported in our previous studies [17, 18].

### Experiment 2: Interpeak Latency of the eCAP

The other aim of this study was to test the hypothesized effect of reduced neural synchrony of the AN on the IPL of the averaged eCAP across 50–100 sweeps, as proposed by Schvartz-Leyzac et al. [21]. Overall, our results do not provide compelling evidence to support the use of the IPL as an indicator of neural synchrony in the AN. The key findings supporting this conclusion are summarized and discussed below.

#### Finding 1

Children with CND, a patient population with substantially fewer but otherwise healthy AN fibers, showed significantly shorter IPLs than both children with ANSD and children with typical SNHL. This suggests that AN fiber count may affect the IPL, with reduced AN fiber leading to shorter IPLs. This finding raises questions about the biological mechanisms that IPL measurements actually reflect.

#### Finding 2

In adult CI users, IPLs increased as the testing electrode moved toward more apical locations. If the IPL exclusively reflects neural synchrony in the AN, these results would suggest that adult CI users have better neural synchrony at the base than at more apical regions of the cochlea. However, this interpretation conflicts with histological findings from human temporal bone studies in CI users showing greater neural degeneration at the base than at the apex of the cochlea [35, 46], as well as neural degenerated patterns observed in age-related hearing loss (e.g., [56]).

#### Finding 3

In adult CI users, IPLs measured at all four electrode locations showed an increasing trend with advancing age. This positive trend reached statistical significance only at the most apical electrode location. These age-related increases are inconsistent with prior evidence of greater AN degeneration at the cochlear base than the apex in older adults [56, 57].

#### Finding 4

No significant associations were found between the IPL and either temporal resolution or speech perception outcomes in children with ANSD or in adult CI users. These results contradict the established relationship between poor neural synchrony in the AN and deficits in temporal resolution and speech perception in noise (e.g., [1]).

#### Finding 5

Consistent with the experimental data, our simulation results demonstrated IPL assessment’s relative lack of sensitivity to the amount of neural synchrony and vulnerability to high measurement error, which further supported this conclusion.

In summary, our data show that the IPL, as a proposed indicator of neural synchrony in the AN, lacks both sensitivity to the relevant underlying neural pathology and predictive value for the resulting auditory perceptual outcomes. Therefore, the current findings do not support the use of IPL as a valid marker of neural synchrony in the AN in CI users.

### Experiment 3: Recording Noise and Sampling Rate

In current CIs, the level of recording noise for eCAP measurements varies across manufacturers and is dependent on the number of sweeps averaged to obtain the eCAP. The noise floor is reported to be approximately 20 μV in older Cochlear™ Nucleus® (e.g., CI24M) implants and 2–5 μV in newer Cochlear™ Nucleus® implants (24RE (CA) or newer) [51] and 20–50 μV [58] in Advanced Bionics devices. For MED-EL devices, the noise floor is frequency dependent, with an estimated overall noise level of 2.6–10 μV [59, 60]. In our analysis of measured recording noise in Experiment 3, we found that the single-sweep noise amplitudes for these adult Cochlear™ Nucleus® users were also frequency dependent—see panel (a) of Fig. 10. As demonstrated by our eCAP results and simulation results, this recording noise increases the unreliability of the IPL over a range of noise levels and the IPLVAR metric at high noise levels. From a theoretical perspective, the reliability of the IPL and IPLVAR measures may be enhanced by eliminating off-frequency noise components via bandpass filtering of eCAPs; this hypothesis could be tested in future work. 

The temporal resolution of peak latency measurements depends on the sampling rate used for eCAP recordings. Higher sampling rates enable more precise identification of response peaks, leading to more accurate IPL and IPLVAR calculations. In current Cochlear™ Nucleus® devices, the sampling rate is 20.492 Hz, resulting in a step size (i.e., the time interval between consecutive samples) of 48.8 μs. This relatively large step size can increase both local and global errors in peak latency estimation. Advanced Bionics devices offer a sampling rate of 56 kHz, corresponding to a finer temporal resolution of approximately 17.85 μs. However, the benefit of this higher resolution is offset by devices’ relatively high noise floor. Based on our simulation results shown in Fig. 11, the high sampling rate (i.e., 1.2 MHz) and the relatively low noise floors of MED-EL devices could improve the accuracy of IPL and IPLVAR assessments. More studies are warranted to test this possibility. Overall, these two hardware-related factors—the noise floor and the sampling rate—limit the reliability of the IPL metric over a range of noise levels and of the IPLVAR metric at high noise levels. Our results, based on a conservative rounding resolution of 1 μV, showed that differences in quantification methods driven by these two hardware-related factors could lead to different outcomes for their associations with some auditory perception measures.

Our simulation results demonstrated that the magnitudes of both IPLVAR and PLV are influenced by recording noise, making the eCAP SNR a potential confounding factor when comparing these metrics across devices from different manufacturers as well as across generations of the same device. Fortunately, the recording noise level can be estimated for each CI patient using eCAP measurements at 0 clinical unit, as demonstrated in our recording noise analysis. Including the estimated noise level as a covariate in statistical analyses where the IPLVAR or the PLV serves as a predictor variable allows for control of recording noise effects on both metrics.

### Study Result Comparisons

In a recent study, Schvartz-Leyzac et al. [21] proposed that decreased neural synchrony should lead to large IPL values. In their study, a significant moderate correlation between the IPL and participant age in data pooled across all electrodes in all adult participants was found, with longer IPLs correlated with more advanced age (see their Fig. 5). Our data, as shown in Fig. 7, showed a similar overall pattern, with the positive trend reaching statistical significance only at the most apical electrode location. As discussed above, this pattern is not consistent with neural degenerated patterns observed in age-related hearing loss (e.g., [56]). At this point, factors accounting for this observed association between age and the IPL remain unknown. It should be noted that our simulation results do not exclude the possibility that the IPL of the mean eCAP waveform can be affected by pathologies that cause a deterministic spreading out of the spike times in a population of AN fibers, leading to a common, broadened eCAP waveform across trials. In these cases, longer IPLs will be observed but they are not due to reduced neural synchrony in the AN. We speculate that the IPL is likely affected by both deterministic changes in the eCAP waveform (common across trials) and intertrial jitter in the eCAP, suggesting that reduced neural synchrony is unlikely to be the only factor underlying the age-related increase in the IPL.

Furthermore, Schvartz-Leyzac et al. [21] reported a negative correlation between AzBio sentence scores at + 10 dB SNR and the IPL (see their Fig. 8), suggesting that longer IPLs were associated with poorer speech perception in noise. In contrast, our study found no such association between the IPL and AzBio sentence scores under any listening condition, regardless of how the IPL was quantified. We identified three key methodological differences in IPL quantification between Schvartz-Leyzac et al. [21] and our study. First, the number of sweeps included in the averaged eCAP responses differed. Schvartz-Leyzac et al. [21] used 50–100 sweeps, whereas we used 400 sweeps, potentially improving the SNR in our data. Second, there were differences in the designated latency windows for identifying the N1 and P2 peaks. Schvartz-Leyzac et al. [21] reported using latency windows of 150–300 μs for N1 and 400–600 μs for P2. However, we noted a discrepancy between these reported time windows and the IPL values shown in their Figures 5 and 6. Based on their defined latency windows, all IPLs should fall within 450 μs or less, yet several IPLs reported in their study exceeded 450 μs, with one even surpassing 500 μs. The reason for this inconsistency is unclear. In our study, broader latency windows were used to better accommodate variabilities in eCAP morphology. Finally, Schvartz-Leyzac et al. [21] did not use any rounding up process to control for the multipoint peak issue. In their analysis, N1 and P2 latencies were measured using the traditional approach [54], in which peak latencies are determined by identifying the minimum and maximum voltage values within predefined latency windows or sample points, without accounting for the noise floor of CI devices.

To determine whether the discrepancies between our findings and those of Schvartz-Leyzac et al. [21] were attributable to methodological differences, we reanalyzed our data using the time windows reported in their Methods section to identify N1 and P2 peak latencies. Specifically, IPLs were recalculated for averaged eCAPs across both the first 50 sweeps and the full set of 400 sweeps, without applying any rounding-up procedure. We then reexamined the associations between IPL and behavioral GDTs, participant age, and AzBio sentence scores obtained under various listening conditions in adult CI users. Supplemental Digital Content 8 shows the association between the IPL, behavioral GDT, and AzBio sentence scores measured in quiet and in noise. For the IPL calculated for the averaged eCAP across 50 sweeps, five participants showed equal IPLs at the two electrode locations with different psychophysical GDTs. Smaller GDTs were measured at the electrodes associated with shorter IPLs in five participants. An opposite pattern was observed in four participants. When using IPLs derived from 400-sweep averages, nine participants had equal IPLs at two electrode locations with different GDTs, effectively ruling out the IPL as a reliable indicator of GDT. In addition, no associations were found between the IPL and AzBio sentence scores under any listening condition, regardless of the number of sweeps used in eCAP averaging. The MLR results supporting this observation are presented in Table 4 and Table B of Supplemental Digital Content 4. Supplemental Digital Content 9 shows IPLs measured at different electrode locations as a function of participant age. These data revealed no correlation between the IPL and age at any electrode location, regardless of the sweep count used in the averaged eCAP. This lack of association is further supported by Pearson correlation results, as indicated in each panel. In summary, our reanalysis showed that the discrepancies between our findings and those of Schvartz-Leyzac et al. [21] cannot be explained by the three methodological differences identified earlier. At present, the factors accounting for this inconsistency remain unknown.

### Comparison Between the IPLVAR and the PLV

Our results, together with findings from previously published studies [17, 18], suggested that both IPLVAR and PLV metrics can serve as indicators of neural synchrony in the AN of CI users. However, each indicator has its own advantages and limitations, which are summarized below.

#### The IPLVAR

##### Advantages

This measure can be applied across CI devices regardless of the manufacturer. The computational method is straightforward and easy to understand. No parameter adjustment is needed when testing patients with CIs from different manufacturers. The IPLVAR is only sensitive to the variability in peak latencies across trials, in contrast to the IPL’s additional dependence on the mean peak latencies (i.e., latency differences that are common across trials).

##### Limitations

As with the IPL, the IPLVAR can be affected by the multipoint peak issue observed in eCAP waveforms, which can compromise precision. In the results presented in this study, the prevalence of multipoint peak occurrences was higher with the IPLVAR method than with the IPL approach. This difference arises because the IPLVAR method is based on single sweeps and therefore does not average out recording noise. Additionally, our simulation results show that the IPLVAR exhibits a systematic shift in its values at moderate levels of recording noise, but its estimates degrade substantially at high noise levels. Consequently, varying recording noise levels across CI devices or generations of the same device may hinder the consistent application of the IPLVAR as a cross-platform metric.

#### The PLV

##### Advantages

The PLV is not affected by the ambiguity introduced by the multipoint peak issue and is affected less by recording noise than the IPL and IPLVAR. The PLV is a well-established concept for assessing neural synchrony in the literature and is only sensitive to variability in the eCAP waveform across trials, not the basic shape of the single-sweep waveform arising from factors that are common across trials, in contrast to the IPL.

##### Limitations

Parameters used in mathematical computations need to be adjusted based on the sampling rate of CI devices from each manufacturer. Current parameter settings are optimized for Cochlear™ Nucleus® devices only. Specific parameters for other CI systems remain undetermined. This limitation complicates direct comparison of PLV results between users of different CI devices.

## Conclusions

The IPL is not a reliable indicator of neural synchrony in the AN. While the IPLVAR can serve as an alternative to the PLV for evaluating AN synchrony in CI users, it is more strongly affected by high levels of eCAP recording noise. Consequently, the PLV emerges as the preferred and more robust metric for quantifying neural synchrony in the AN of CI users.

## Supplementary Information

Below is the link to the electronic supplementary material.

ESM1 (DOCX 247 KB)

ESM2 (DOCX 257 KB)

ESM3 (DOCX 18.2 KB)

ESM4 (DOCX 300 KB)

ESM5 (DOCX 157 KB)

ESM6 (DOCX 178 KB)

ESM7 (DOCX 445 KB)

ESM8 (DOCX 192 KB)

ESM9 (DOCX 17.5 KB)

## Data Availability

The data that support the findings of this study are available from the authors upon reasonable request with permissions from The Ohio State University and the University of North Carolina at Chapel Hill.

## Code Availability

The programming code used in this study is available from the authors upon reasonable request with permission from The Ohio State University.
