# Supplementary material for: Evaluating the N1-P2 Interpeak Latency of the eCAP and Its Intertrial Variability as Potential Indicators of Neural Synchrony in the Auditory Nerve of Cochlear Implant Users
Source: J Assoc Res Otolaryngol. 2026 May 11;27(4):583–609. doi: 10.1007/s10162-026-01051-1 (PMC13428357; doi:10.1007/s10162-026-01051-1)
Supplement: Supplementary file 1 [file 10162_2026_1051_MOESM1_ESM.docx]

**Figure A**. A Chi-square goodness-of-fit test with a 1% significance level was performed on the IPL distributions from the 107 electrodes in the Gao et al. [18] dataset using the function chi2gof from the MATLAB Statistics and Machine Learning Toolbox. 34 out of 107 IPL distributions were found to be well fit by a gamma distribution. An equal number of IPL distributions were found to be well fit by a normal distribution, however a histogram of skewness values for the IPL distributions (subfigure a) shows that there are more IPL distributions with positive (right) skew than zero skew or negative (left) skew, such that the gamma distribution is preferable to the normal distribution for modeling the IPL distributions. Subfigures b and c show analyses of the gamma distribution fits for two example cases that are representative of distributions that failed the Chi-square goodness-of-fit test. The top, middle, and bottom rows in each subfigure show the gamma distribution fit to the N1 latency, P2 latency, and IPL, respectively. The left columns show the latency histograms with gamma-distribution fits in red, while the right columns show plots of the quantiles of the measured latencies versus the theoretical quantile values from the fitted gamma distribution. For electrode 15 of A008R (subplot b), the N1 and P2 latency distributions are both greatly impacted by the sampling rate of the eCAP measurements, such that neither of these latency distributions nor the IPL distribution pass the goodness-of-fit test. In contrast, electrode 15 of A017R (subplot c) has a P2 latency distribution that is well fit by a gamma distribution, but the N1 latency distribution is once again greatly impacted by the eCAP sampling rate, such that the IPL distribution narrowly fails the goodness-of-fit test with a 1% significance level. Thus, despite these types of IPL distributions failing the goodness-of-fit tests, qualitatively the gamma distribution appears to be a reasonable to use for the simulations in this study.
