# Supplementary material for: Evaluating the N1-P2 Interpeak Latency of the eCAP and Its Intertrial Variability as Potential Indicators of Neural Synchrony in the Auditory Nerve of Cochlear Implant Users
Source: J Assoc Res Otolaryngol. 2026 May 11;27(4):583–609. doi: 10.1007/s10162-026-01051-1 (PMC13428357; doi:10.1007/s10162-026-01051-1)
Supplement: Supplementary file 2 [file 10162_2026_1051_MOESM2_ESM.docx]

**Figure B**. Gamma distributions were fitted to IPL distributions from the simulated eCAPs. Panel a shows histograms of the *a* (shape) and *b* (scale) gamma distribution parameters from these simulations over the full range of jitter factors 0.0 to 2.0. Comparing these histograms to those obtained from the human data (Fig. 2b,c), it can be observed that they are fairly consistent except for the higher proportion of very low scale parameter values (30 occurrences in the first histogram bin) and the range of *b* (scale) parameters for the simulations is less than that for the human data. This is to be expected, because the simulations are not aimed at explaining all of the IPL distributions in the data but rather just a range of distributions with different degrees of spread around an average IPL value for the population. Panel b shows histograms of the estimated gamma distribution parameters from simulations only over the range of jitter factors from 0.3 to 2.0. In this case the simulation histograms better match the human data histograms, indicating that it is the jitter factors in the range 0.0 to 0.2 that are producing the very low scale parameters values. However, it is useful to include such low jitter factors in the simulations to demonstrate the theoretical range of IPL, IPLVAR, and PLV values that could be obtained when eCAP sampling rate and recording noise are not limiting factors.
