# Supplementary material for: Evaluating the N1-P2 Interpeak Latency of the eCAP and Its Intertrial Variability as Potential Indicators of Neural Synchrony in the Auditory Nerve of Cochlear Implant Users
Source: J Assoc Res Otolaryngol. 2026 May 11;27(4):583–609. doi: 10.1007/s10162-026-01051-1 (PMC13428357; doi:10.1007/s10162-026-01051-1)
Supplement: Supplementary file 3 [file 10162_2026_1051_MOESM3_ESM.docx]

**Table B**. Results of multiple linear regressions (MLRs) for assessing the associations between AzBio sentence scores measured in different listening conditions and the interpeak latency (IPL) results quantified in different ways without controlling the age effect. SNR: signal to noise ratio; SE: standard error.

| IPL Calculation Method | Listening Environment | Unstandardized *β (SE)* | *t* | *p* | *R^2^* |
| --- | --- | --- | --- | --- | --- |
| Min | Quiet | -0.0368 (0.0701) | -0.524 | .604 | 0.0105 |
|  | Noise, SNR +10 dB | -0.0274 (0.0959) | -0.285 | .778 | 0.0031 |
|  | Noise, SNR +5 dB | -0.0282 (0.0732) | -0.385 | .704 | 0.0057 |
| Max | Quiet | -0.0558 (0.0699) | -0.798 | .432 | 0.0239 |
|  | Noise, SNR +10 dB | -0.0468 (0.0960) | -0.488 | .630 | 0.0091 |
|  | Noise, SNR +5 dB | -0.0519 (0.0730) | -0.711 | .483 | 0.0191 |
| Average | Quiet | -0.0473 (0.0708) | -0.668 | .510 | 0.0169 |
|  | Noise, SNR +10 dB | -0.0379 (0.0970) | -0.390 | .699 | 0.0058 |
|  | Noise, SNR +5 dB | -0.0409 (0.0739) | -0.553 | .585 | 0.0116 |
| Schvartz-Leyzac et al. (2025) | Quiet | 0.1874 (0.2465) | 0.760 | .454 | 0.0218 |
|  | Noise, SNR +10 dB | 0.0015 (0.3396) | 0.005 | .996 | < 0.0001 |
|  | Noise, SNR +5 dB | -0.0646 (0.2592) | -0.249 | .805 | 0.0024 |
| Schvartz-Leyzac et al. (2025)  50 Sweeps | Quiet | 0.3586 (0.2009) | 1.785 | .086 | 0.1091 |
|  | Noise, SNR +10 dB | 0.3219 (0.2831) | 1.137 | .266 | 0.0474 |
|  | Noise, SNR +5 dB | 0.0877 (0.2210) | 0.397 | .695 | 0.0060 |
