# Supplementary material for: Evaluating the N1-P2 Interpeak Latency of the eCAP and Its Intertrial Variability as Potential Indicators of Neural Synchrony in the Auditory Nerve of Cochlear Implant Users
Source: J Assoc Res Otolaryngol. 2026 May 11;27(4):583–609. doi: 10.1007/s10162-026-01051-1 (PMC13428357; doi:10.1007/s10162-026-01051-1)
Supplement: Supplementary file 4 [file 10162_2026_1051_MOESM4_ESM.docx]

**Figure C**. Panel (a) shows the simulated recording noise magnitude-frequency spectra for Figure 11 (h)–(m) and panels (b)–(g) of this figure. The IPL, IPLVAR, and PLV simulation results as a function of SNR for the sampling frequencies of 100 kHz—panels (b)–(d)—and 56 kHz—panels (e)–(g)—show behaviors that are intermediate between the 500 kHz and 20.492 kHz simulation results shown in Figure 11.
