# Supplementary material for: Evaluating the N1-P2 Interpeak Latency of the eCAP and Its Intertrial Variability as Potential Indicators of Neural Synchrony in the Auditory Nerve of Cochlear Implant Users
Source: J Assoc Res Otolaryngol. 2026 May 11;27(4):583–609. doi: 10.1007/s10162-026-01051-1 (PMC13428357; doi:10.1007/s10162-026-01051-1)
Supplement: Supplementary file 5 [file 10162_2026_1051_MOESM5_ESM.docx]

**Figure D**. Additional eCAP simulations were performed with the interpeak latency (IPL) distribution being a normal distribution rather than a gamma distribution. All simulations were performed at a sampling rate of 500 kHz without simulated recording noise. The baseline normal distribution (thick black curve in panel a) had a mean and standard deviation matching those of the baseline gamma distribution from the main simulations, and temporal scaling of the normal distribution around its mean as a function of the jitter factor was applied in the same manner as for the gamma distribution (see panel a). The IPL, intertrial variability of the IPL (IPLVAR), and phase locking value (PLV) versus jitter factor relationships for these normal-distribution simulations (panels b–d) were very similar to those for the gamma distribution (Figure 11).
