# Supplementary material for: Evaluating the N1-P2 Interpeak Latency of the eCAP and Its Intertrial Variability as Potential Indicators of Neural Synchrony in the Auditory Nerve of Cochlear Implant Users
Source: J Assoc Res Otolaryngol. 2026 May 11;27(4):583–609. doi: 10.1007/s10162-026-01051-1 (PMC13428357; doi:10.1007/s10162-026-01051-1)
Supplement: Supplementary file 6 [file 10162_2026_1051_MOESM6_ESM.docx]

**Figure E**. Additional simulations were performed with template electrically evoked compound action potential (eCAP) waveforms created from four other electrodes in the third dataset that had high phase locking value (PLV) values and relatively high signal to noise ratios (SNRs). All simulations were performed at a sampling rate of 500 kHz without simulated recording noise. Panel a shows these four alternative template waveforms. Despite these waveforms having substantial differences from the template eCAP used in the main simulations, the interpeak latency (IPL), intertrial variability of the IPL (IPLVAR), and PLV versus jitter factor relationships for these additional simulations (panels b–d) were qualitatively similar to those for the standard template (Figure 11). Two of these template waveforms (e9 of A080L and e21 of A087L) caused a more pronounced initial drop in the PLV as the jitter factor increases from 0 to 0.1 (see the green and purple curves in panel d), and the other two template waveforms (e15 and e9 of A008R) produced a non-monotonic relationship between the IPL and the jitter factor (see the blue and orange curves in panel b).
