# Supplementary material for: Evaluating the N1-P2 Interpeak Latency of the eCAP and Its Intertrial Variability as Potential Indicators of Neural Synchrony in the Auditory Nerve of Cochlear Implant Users
Source: J Assoc Res Otolaryngol. 2026 May 11;27(4):583–609. doi: 10.1007/s10162-026-01051-1 (PMC13428357; doi:10.1007/s10162-026-01051-1)
Supplement: Supplementary file 8 [file 10162_2026_1051_MOESM8_ESM.docx]

**Figure G**. The associations between interpeak latencies quantified using the designated latency windows reported in Schvartz-Leyzac et al. (2025) and participant age. Panels listed in the top and the bottom rows represent the results averaged across 50 and 400 sweeps, respectively. Each panel represents the results measured at one electrode location. Each symbol indicates the result from one participant. Solid lines represent the results of linear regression. The results of Pearson correlation tests are indicated in each panel.
